# Creeping fig (*Ficus pumila* L.) polysaccharides: a review of extraction methods, structural characteristics, biological activities, and emerging applications in medicine

**DOI:** 10.3389/fphar.2026.1921886

**Published:** 2026-08-25

**Authors:** Dan Xie, Wei Chen, Shuo Niu, Yu Sun, He Jiang

**Affiliations:** 1 The Second Affiliated Hospital of Heilongjiang University of Chinese Medicine, Harbin, China; 2 Heilongjiang Provincial Hospital, Harbin, China

**Keywords:** biological activities, *Ficus pumila* L., medical applications, polysaccharides, structure–activity relationships

## Abstract

Creeping fig (*Ficus pumila* L.) is a traditional plant with both edible and medicinal value, and its achenes can spontaneously form gels at room temperature. As important bioactive macromolecules in creeping fig, polysaccharides have attracted increasing attention due to their biological activities and unique gelling properties. However, current studies on creeping fig polysaccharides remain scattered, and there is still a lack of systematic integration of their extraction and purification methods, structural characteristics, biological activities, structure-activity relationships, hydrogel properties and potential applications. This review summarizes recent progress on creeping fig polysaccharides, with particular attention to the relationship between structural characteristics, biological activities and hydrogel properties, as well as the current evidence supporting their potential applications. Existing studies have shown that creeping fig polysaccharides possess multiple biological activities, including hypoglycemic, anti-colitis, anti-obesity, immunomodulatory and anti-cellular senescence activities, among which inflammation regulation and gut microbiota regulation are frequently involved. In terms of structural characteristics, molecular weight, monosaccharide composition, galacturonic acid content, degree of methylesterification and degree of acetylation are important factors affecting their physicochemical properties, biological activities and hydrogel formation. Creeping fig polysaccharides with low-methoxyl pectin characteristics can form calcium-crosslinked hydrogels, showing potential applications in tissue repair, biolubrication and ultrasound-assisted imaging, although these applications still require further experimental validation. This review aims to provide a systematic and critical reference for the further development of creeping fig polysaccharides in functional food, pharmacology and medicine.

## 1 Introduction

**FIGURE 1:**
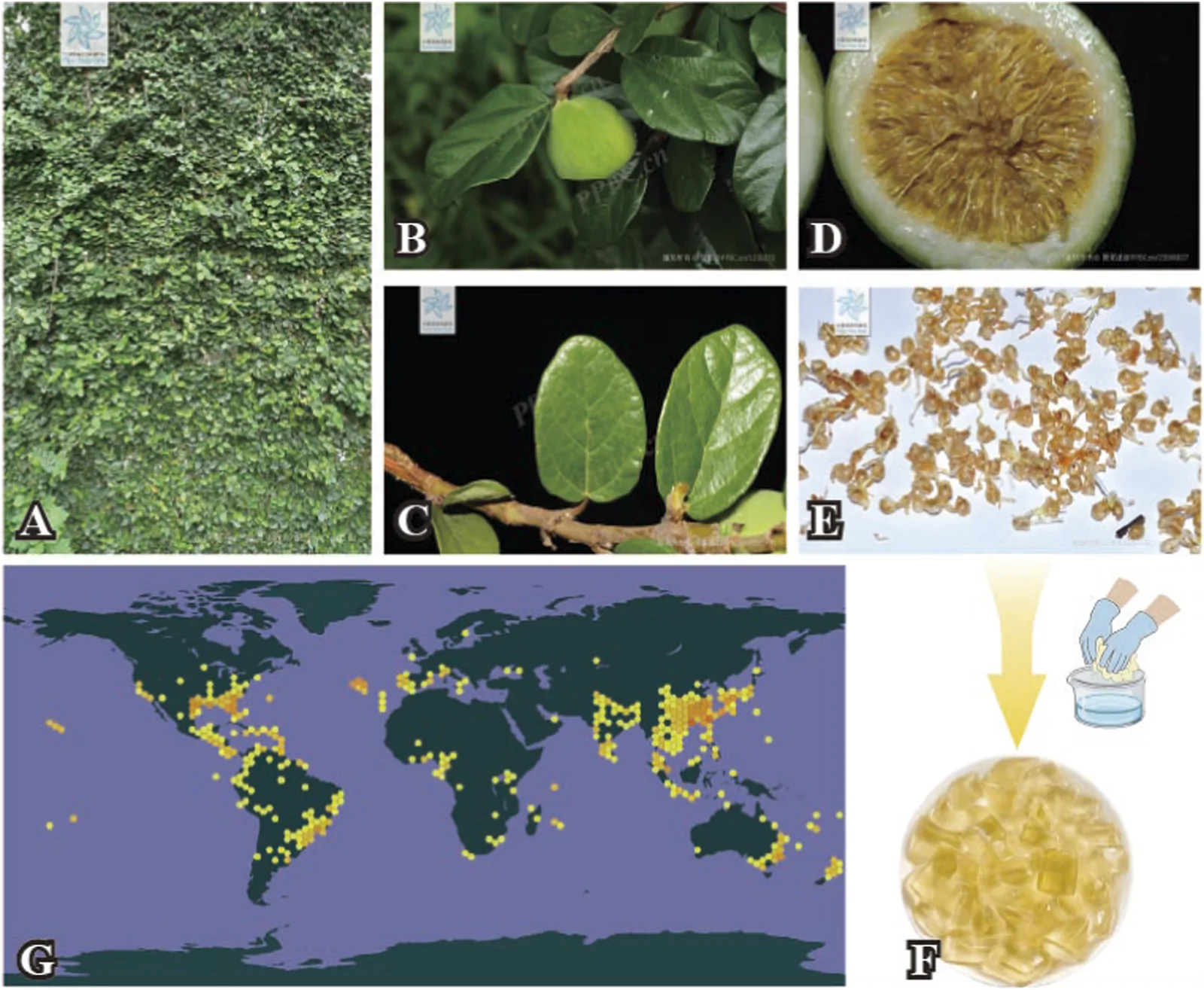
Morphological characteristics and distribution of creeping fig. **(A)** The plant; **(B)** syconium; **(C)** leaves; **(D)** cross-section of the syconium; **(E)** achenes; (Source: https://www.iplant.cn); **(F)** creeping fig jelly; **(G)** worldwide distribution. (Source: https://www.gbif.org).

Creeping fig (*Ficus pumila* L.) is a perennial evergreen creeping plant of *Ficus* in Moraceae (Salehi et al., 2021). It is mainly distributed in subtropical and tropical regions such as China, Japan, and South Korea (Gonda et al., 2025) (Figure 1). Creeping fig relies on aerial roots to grow on walls, rocks, or trunks. Its syconium is nearly spherical or obovate and contains a large number of achenes after maturity (Figure 1). The surface of achenes is covered with a layer of mucilage, which is a significant morphological feature of creeping fig different from other common edible plants (Luo et al., 2024). It is by virtue of this unique structure that the achene of the creeping fig becomes its main edible part. After the achene of creeping fig was rubbed and extracted, the mucilage on the surface could spontaneously form a gel at room temperature and then processed into the traditional summer dessert “Liangfen” for consumption. This unique processing and eating method has a history of hundreds of years in southern China and Southeast Asia, which constitutes an important traditional edible basis for creeping fig (Liang et al., 2012).

In addition to its traditional edible value, creeping fig has also received extensive attention due to its rich nutritional metabolites and diverse medicinal values. From the perspective of nutrition, creeping fig is rich in carbohydrates, vitamins, and minerals, and the fat content is low. Among them, the mucilaginous metabolite on the surface of achenes is particularly distinctive. This metabolite can form a gel in water. As a natural water-soluble dietary fiber, it helps to regulate intestinal function, increase satiety, and meet the current consumer demand for healthy natural food (Chen et al., 2024). Creeping fig also has a long history of use in traditional empirical medicinal practice. Different parts of the plant have been used for different purposes. The stems and leaves are traditionally used for dispelling wind and dampness, promoting blood circulation, dredging collaterals, detoxification and detumescence, and are commonly associated with rheumatic arthralgia, sciatica, diarrhea, stranguria, edema, sore throat, carbuncles, and traumatic injury (Liao et al., 2012; Suzuki et al., 2021). The roots are mainly used for dispelling wind and dampness, relaxing tendons and dredging collaterals, and are associated with rheumatic arthralgia, sciatica, lumbar muscle strain, edema, chronic nephritis, chronic enteritis, and traumatic injury. The latex has been used externally for relieving itching and treating skin-related disorders. The syconia are traditionally considered to have kidney-tonifying, dampness-removing, blood-activating, lactation-promoting, and detoxifying effects, and have been used for seminal emission, impotence, stranguria, chronic dysentery, hemorrhoidal bleeding, prolapse, amenorrhea, hernia, insufficient lactation, sore throat, carbuncles, and scabies (Kitajima et al., 2000). With the development of modern pharmacological research, antibacterial, antioxidant, hypoglycemic, and other biological activities of creeping fig have gradually been reported (Deepa et al., 2018; Suzuki et al., 2021; Qi et al., 2021). These nutritional and medicinal characteristics have attracted increasing attention from researchers and have gradually made creeping fig an important natural source for the research and development of functional metabolites.

With the deepening of research on creeping fig, researchers have found that it contains flavonoids, triterpenoids, sterols, phenolic acids, polysaccharides, and other bioactive metabolites, among which polysaccharides are an important class of bioactive macromolecules (Leong et al., 2008; Xiao et al., 2024). Studies have shown that creeping fig polysaccharides, especially pectin polysaccharides, are the main metabolites of the mucilage layer on the surface of creeping fig achenes, which are closely related to the natural gel properties of creeping fig. In addition, creeping fig polysaccharide also has many biological activities, such as immune regulation, regulation of intestinal flora, and delaying cell senescence, and its health effects have gradually attracted attention. Therefore, creeping fig polysaccharide is not only an important material basis for its various biological activities and gel properties, but also a kind of natural resource with high development potential.

Although the research on creeping fig polysaccharide is increasing, the existing information is still relatively scattered. Most studies only focus on single aspects such as extraction, structures or activities, and the links between various aspects still lack systematic integration and summarization. Throughout the existing literature, there is no specific review of creeping fig polysaccharide. Therefore, this review systematically summarized the extraction and purification methods of creeping fig polysaccharide for the first time, clarified its structural characteristics, biological activities and structure-activity relationships, and focused on its hydrogel properties and application potential in the medical field, such as tissue repair, biolubrication, and ultrasound imaging. Through the integration of these studies, this review aims to provide a reference for further research and development of creeping fig polysaccharide in the medical and health field.

## 2 Methodology

A structured literature search was conducted to collect studies related to creeping fig polysaccharides published between 2000 and 2026. The databases searched included PubMed, ScienceDirect, and Google Scholar. The search terms were combined using Boolean operators and mainly included plant-related terms and polysaccharide-related terms, such as (“*F. pumila*” OR “creeping fig”) AND (“polysaccharide” OR “pectin” OR “hydrogel”). Before literature screening, the botanical name of creeping fig was checked according to Flora of China and World Flora Online to ensure taxonomic accuracy. Studies were included if they focused on creeping fig polysaccharides or pectic polysaccharides and provided information on extraction, purification, structural characterization, physicochemical properties, biological activities, hydrogel formation or potential applications. Studies were excluded if they were unrelated to creeping fig, focused only on non-polysaccharide metabolites, lacked extractable information on polysaccharides or pectin, or were duplicate or inaccessible records. Studies on *F. pumila* var. *awkeotsang* were not included in the main analysis because they represent a closely related but taxonomically distinct material outside the scope of this review. Finally, 16 studies were included and classified according to extraction and purification methods, structural features, biological effects, hydrogel properties, and application-related evidence.

During literature screening and data extraction, terminological inconsistencies were found in the description of raw material parts, mainly reflected in the mixed use of “fruit”, “syconium”, “seed” and “achene”. From the perspective of plant anatomy, the edible structure of creeping fig is a syconium, which is a hollow enclosed inflorescence. The small dry fruits located inside the syconium are achenes, and each achene contains a true seed. The botanical distinction between the syconium and the enclosed achenes is illustrated in Figure 1. However, many original studies did not provide sufficient anatomical details to determine whether “fruit” referred strictly to the syconium and whether “seed” referred strictly to the achene or the true seed. Therefore, the original terminology was retained in tables and in descriptions of specific experimental materials, whereas standardized botanical terms such as “syconium” and “achene” were used in the integrated discussion.

## 3 Extraction and purification of creeping fig polysaccharides

**FIGURE 2:**
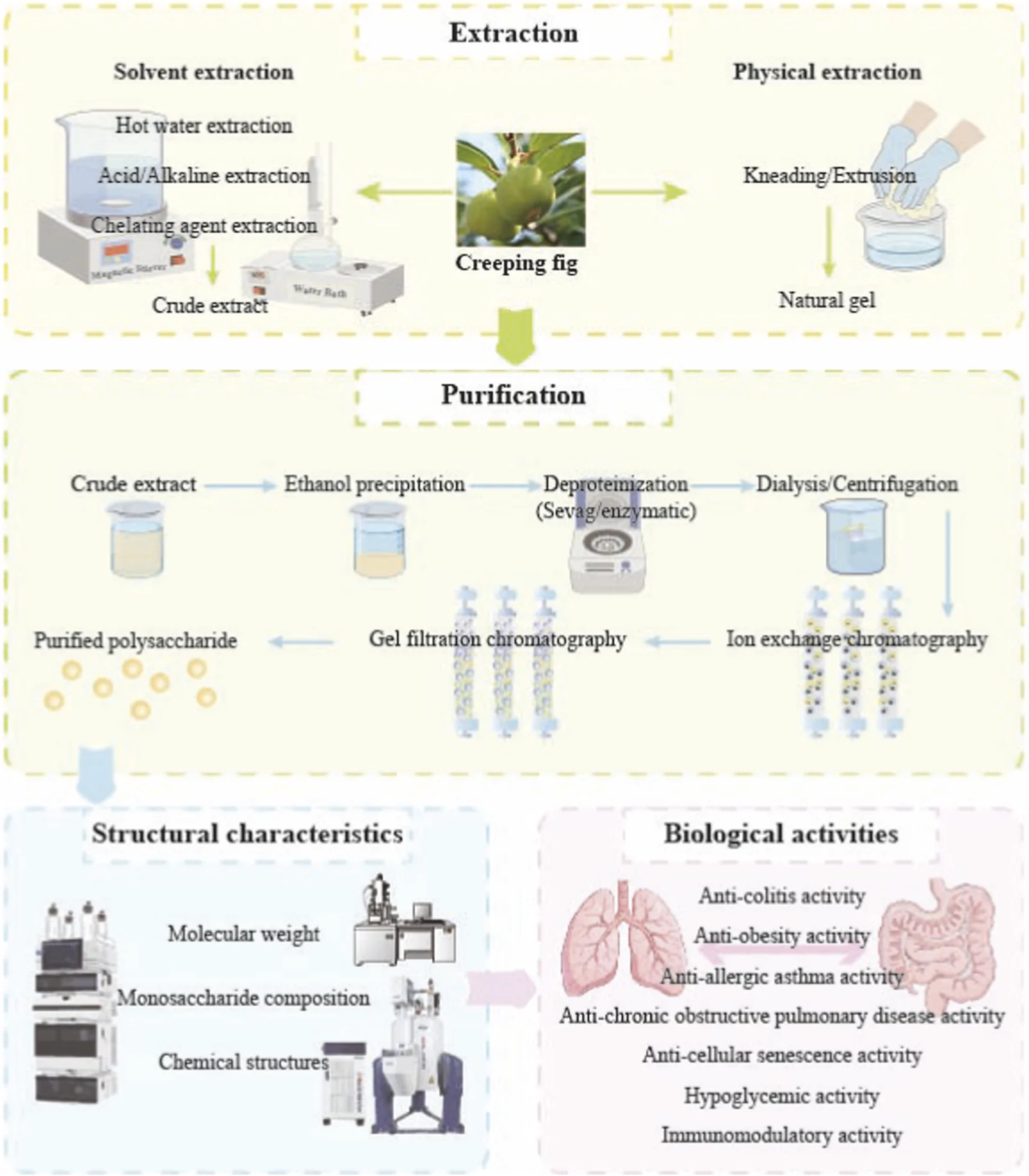
Flowchart of the extraction, purification, structural characteristics, and biological activities of creeping fig polysaccharides.

The extraction and purification of polysaccharides are the premise of studying their structures and bioactivities (Yang et al., 2015; Mazarei et al., 2017). The selection of extraction method and purification process will directly affect the yield, purity, structures, and activities of the obtained polysaccharides. A summary of the extraction and purification methods reported for creeping fig polysaccharides is provided in Table 1. Figure 2 summarizes the workflow for extraction, purification, structural characteristics, and biological activities of creeping fig polysaccharides.

**TABLE 1:** Extraction and purification of creeping fig polysaccharides.

Source	Extraction				Purification		Ref
	Total polysaccharide	Extraction method	Extraction parameters	Yield	Polysaccharide fraction	Purification method	
Leaves	Crude extract	Hot water extraction	2 × 2 h; 100 °C; 1: 10	N/A	FPP-C	Ethanol precipitation, enzymatic treatment, dialysis	Chen et al. (2026)
	Crude extract	Hot water extraction	2 h→1.5 h; 90 °C; 1: 10→1: 8	N/A	FPLP-M	Ethanol precipitation, papain deproteinization, dialysis	Mo et al. (2026)
Fruit hulls	FPP-W1	Hot water extraction	3 × 3 h; 100 °C; 1: 10	4.85%	FPW-W	DEAE-sepharose fast flow, Superdex^TM^ G-200, dialysis	Wu et al. (2020a)
Fruits	Crude extract	Hot water extraction	2 × 2 h; 100 °C; 1: 10	N/A	FPLP-C	Ethanol precipitation, enzymatic treatment, dialysis	Chen et al. (2026)
	Crude extract	Hot water extraction	3 × 3 h; 100 °C; 1: 10	N/A	FPP-K	Ethanol precipitation, deproteinization with papain, dialysis	Kong et al. (2024)
	FPP-W2	Hot water extraction	3 × 3 h; 100 °C; 1: 10	N/A	FPLP-W	DEAE-sepharose fast flow, Superdex^TM^G-75, ethanol precipitation, deproteinization with papain, ion exchange chromatography, gel filtration chromatography, dialysis	Wu et al. (2017), Wu et al. (2020b), Chen et al. (2022)
	FPLP/DM25/DA2	Hot water extraction	3 × 3 h; 100 °C; 1: 10	0.81%	N/A	Dialysis	Chen et al. (2024)
	Chemically Modified FPLP	De-methylesterification	0.5 h; 4 °C	N/A	DM3		
		Methylesterification	0.5 h; 20 °C–25 °C	N/A	DM54		
		Methylesterification	14 h; 20 °C–25 °C	N/A	DM94		
		Acetylation	0.5 h; 60 °C	N/A	DA23		
	Crude extract	Hot water extraction	2–3 h; 90 °C–95 °C; 1: 15	N/A	FPP-Y	Ethanol precipitation, deproteinization with Sevag reagent, dialysis	Ye et al. (2024), Ye et al. (2025)
	Crude extract	Hot water extraction	2 h→1.5 h; 90 °C; 1: 10→1: 8	N/A	FPP-M	Ethanol precipitation, papain deproteinization, dialysis	Mo et al. (2026)
Seeds	Crude extract	Water extraction	0.5 h; 20 °C–25 °C; 1: 20	N/A	FPL pectin	Ethanol precipitation, fractional precipitation	Luo et al. (2024)
	Crude extract	Water extraction	0.5 h; 20 °C–25 °C; 1: 20	N/A	CFSP	Ethanol fractionation, centrifugation	Liu et al. (2021)
	Pectin-C	Water extraction	0.5 h; 20 °C–25 °C; 1: 20	N/A	N/A	Ethanol fractionation	Chen et al. (2021)
	WEP	Water extraction	0.5 × 3 h; 25 °C; 1: 20	5.25%	N/A	Ethanol precipitation	Liang et al. (2012)
	ChEP	Chelating agent extraction	0.5 × 3 h; 25 °C; 1: 20	5.25%–6.07%	N/A	Ethanol precipitation, dialysis	
	HEP	Acid extraction	0.5 × 3 h; 85 °C; 1: 20	6.07%	N/A	Ethanol precipitation	
​	WEP	Water extraction	0.5 × 3 h; 25 °C; 1: 20	N/A	WEP-03	Ethanol precipitation	Wang et al. (2019)
					WEP-04		
Stems	Crude extract	Hot water extraction	2 h→1.5 h; 90 °C; 1: 10→1: 8	N/A	FPSP	Ethanol precipitation, papain deproteinization, dialysis	Mo et al. (2026)

N/A: information was not available.

### 3.1 Extraction methods

Extraction is the primary step to release polysaccharides from plant raw materials (Luan et al., 2024; Sun et al., 2025). The choice of extraction method mainly depends on the raw material part and the existence state of the target polysaccharide (Shi, 2016). Hot water extraction is the main extraction method of polysaccharides from creeping fig syconium, fruit hull, stem, and leaf. This method uses water as a solvent to promote the loosening of cell wall structures and accelerate the diffusion of polysaccharides into the solvent by heating (Khalil and Lukasiewicz, 2024). It has the advantages of simple operation, low cost, and no organic solvent residue (Wang et al., 2026). In the study of creeping fig polysaccharide, hot water extraction is usually carried out at 90 °C–100 °C, the ratio of material to liquid is mostly 1: 10, and the extraction is repeated 2–3 times to improve the yield. Wu et al. (2020a) obtained FPW-W from the fruit hull, Wu et al. (2017) obtained FPLP-W from creeping fig syconium by hot water extraction, Mo et al. (2026) further extracted FPLP-M, FPSP and FPP-M from leaves, stems and syconium. Hot water extraction is suitable for the acquisition of water-soluble polysaccharides, which is a conventional method for the preparation of polysaccharides from creeping fig. Water extraction at room temperature is mainly used for the preparation of pectin from achenes of creeping fig (Liang et al., 2012). The mucilage layer on the surface of creeping fig achene is rich in pectin substances, and its dissolution can be achieved without high temperature. Therefore, this kind of pectin is usually extracted with water as solvent at 20 °C–25 °C, and the solid-liquid ratio is mostly 1: 20. This mild extraction condition can effectively avoid the destruction of pectin structures at high temperature, thus retaining its natural gel properties. Liang et al. (2012) obtained water-soluble pectin polysaccharide WEP by water extraction at room temperature. Wang et al. (2019) further isolated pectin polysaccharides such as WEP-0.3 and WEP-0.4 based on WEP. In addition to water extraction, creeping fig polysaccharides can also be obtained by chelating agent extraction and acid extraction. Chelating agent extraction uses chelating agent to bind calcium ions in the cell wall to promote the dissolution of pectin bound to calcium cross-linking, and the yield of polysaccharide (ChEP) is about 5.25%–6.07% (Liang et al., 2012). Acid extraction was carried out under acidic conditions at 85 °C, which could further promote the hydrolysis and release of bound pectin, and the yield of polysaccharide (HEP) was about 6.07%. It can be seen that these two methods can improve the yield of pectin.

In general, the extraction of creeping fig polysaccharides is still dominated by hot water extraction and room-temperature water extraction. Among them, hot water extraction is mainly suitable for polysaccharides from syconium, fruit hulls, stems and leaves, while room-temperature water extraction, chelating agent extraction and acid extraction are mainly used to obtain pectin from achenes. For creeping fig polysaccharides, water-based extraction itself has certain advantages because it is mild, simple and solvent-safe, and is more suitable for preserving the pectic structure, molecular weight (Mw) distribution and natural gel-forming properties. Green or intensified extraction methods, such as ultrasound-assisted extraction, microwave-assisted extraction and deep eutectic solvent-based extraction, have been applied to several plant polysaccharides, including tea polysaccharides, marshmallow root polysaccharides and *Cercis chinensis* bark polysaccharides, showing potential advantages in improving extraction efficiency or modifying physicochemical properties (Hashemifesharaki et al., 2020; Xia et al., 2023; Shu et al., 2025). These studies suggest that such methods may also provide methodological references for creeping fig polysaccharides. However, considering the pectic structure and natural gel-forming properties of creeping fig polysaccharides, future extraction studies should not focus only on yield improvement. The effects of different extraction methods on Mw, galacturonic acid (GalA) content, chain conformation and hydrogel-forming ability should also be evaluated. Therefore, the selection of extraction methods is closely related not only to polysaccharide recovery, but also to subsequent structural characterization, activity evaluation and hydrogel construction.

### 3.2 Purification methods

Purification is an important prerequisite for obtaining homogeneous polysaccharide metabolites and carrying out fine structure analysis (Ji et al., 2018; Wang et al., 2018). The polysaccharides extracted from creeping fig are crude polysaccharides, which are often accompanied by proteins, pigments, inorganic salts and other small molecular impurities. Therefore, it is necessary to improve the purity of the samples by various purification methods. Alcohol precipitation and graded alcohol precipitation are the basic steps for the purification of creeping fig polysaccharide (Fernando et al., 2020; Fan et al., 2022). The preliminary separation of polysaccharides and small molecular impurities can be achieved by using the characteristics of the reduced solubility of polysaccharides in high concentration ethanol (Xu et al., 2014). In the study of achene-derived pectin, the graded alcohol precipitation method is often used to obtain different pectin metabolites. For example, Wang et al. (2019) obtained metabolites such as WEP-0.3 and WEP-0.4 from WEP by ethanol fractional precipitation, while Liu et al. (2021) obtained CFSP by fractional alcohol precipitation, which provided a basis for subsequent structures and gel properties. Deproteinization is used to remove the coexisting protein impurities in crude polysaccharides (Zeng et al., 2019; Yang et al., 2022). The commonly used methods in the study of creeping fig polysaccharide include papain enzymatic hydrolysis and the Sevag method. The former uses protease to degrade proteins, which has the advantages of mild conditions and less damage to the structures of polysaccharides (Lin et al., 2023). The latter is separated by protein denaturation and precipitation with organic reagents (Zhao et al., 2024). In recent years, the papain method has been widely used. Ye et al. (2024), Ye et al. (2025) and Mo et al. (2026) used this method to deproteinize crude polysaccharides. Dialysis is a commonly used auxiliary step in the purification process of creeping fig polysaccharide, which is mainly used to remove inorganic salts, monosaccharides and other low Mw impurities (Bagnol et al., 2022). Through the selective retention of the dialysis membrane, macromolecular polysaccharides were retained, while small molecular impurities gradually diffused into the dialysis solution, thereby further improving the purity of the sample (Jaros et al., 2018). Column chromatography is a step for further grading and refining polysaccharides (Qi et al., 2021). Ion exchange chromatography and gel filtration chromatography are the two most widely used technologies. Among them, DEAE-Sepharose ion exchange chromatography can separate neutral and acidic polysaccharides according to the charge difference of polysaccharides, while Superdex G-75 or G-200 gel filtration chromatography can further purify polysaccharide metabolites according to molecular size (Long et al., 2021). Wu et al. (2017) and Wu et al. (2020a) obtained homogeneous polysaccharide metabolites such as FPLP-W and FPW-W by DEAE-Sepharose and Superdex gel filtration, respectively, which significantly improved the purity of the samples and laid a foundation for subsequent structural identification and activity evaluation.

With the improvement of purification degree, the Mw distribution, monosaccharide composition and other structural characteristics of creeping fig polysaccharide can be more accurately characterized, so as to provide a reliable basis for elucidating its biological activities.

## 4 Structural characteristics of creeping fig polysaccharides

**FIGURE 3:**
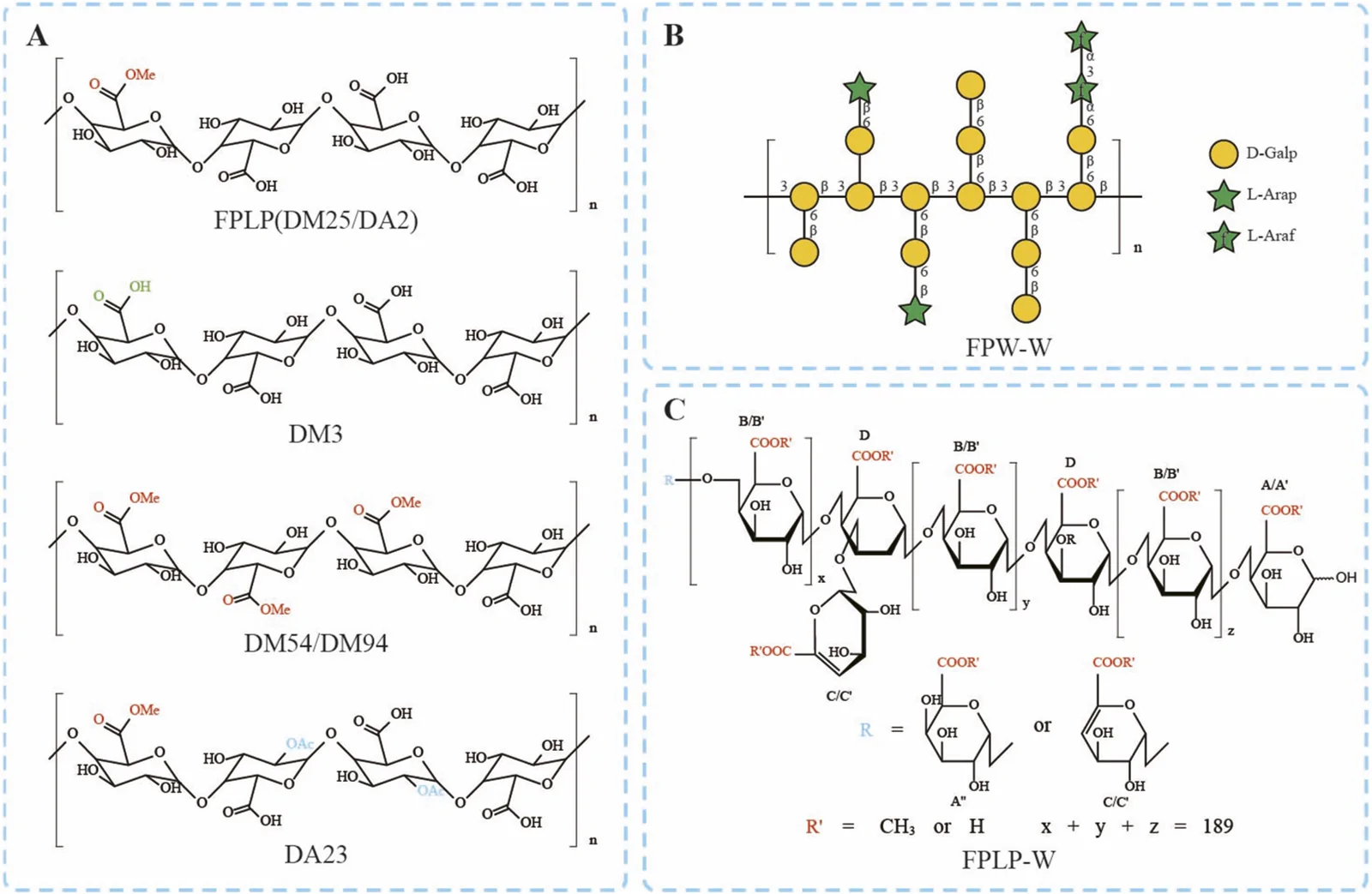
Chemical structures of creeping fig polysaccharides. **(A)** FPLP and its derivatives with different DM and DA (DM3, DM54/DM94, DA23); **(B)** FPW-W; **(C)** FPLP-W.

The structures of polysaccharides are the basis of their physicochemical properties and biological activities (Silva et al., 2021; Luan et al., 2022). The polysaccharides prepared by different methods from different parts of creeping fig showed significant differences in Mw, monosaccharide composition and chemical structures. At present, researchers mainly use high performance gel permeation chromatography (HPGPC), ion chromatography (HPAEC), gas chromatography-mass spectrometry (GC-MS), Fourier transform infrared spectroscopy (FT-IR) and nuclear magnetic resonance (NMR) to characterize the structures of creeping fig polysaccharide. The structural characteristics are shown in Figure 3. This section integrates the existing research results (core data are shown in Table 2) to systematically elucidate the structural characteristics of creeping fig polysaccharide.

**TABLE 2:** Structural characteristics of creeping fig polysaccharides.

Source	Name	Molecular weights	Monosaccharide composition	Structure and properties		Analytical technique	Ref
Leaves	FPLP-M	Mw = 202.65 kDa	Fuc: Rha: Ara: Gal: Glc: Xyl: Man: GalA: GlcA = 0.44: 0.19: 0.23: 0.87: 1.00: 0.22: 0.28: 0.06: 0.08	FT-IR: 3,414 cm^−1^: O–H stretching; 2,974 cm^−1^: C–H stretching; 1,609 cm^−1^: COO− asymmetric stretching; 1,075 cm^−1^: neutral sugar side-chain vibration; 1740 cm^−1^: no obvious absorption peak Type: neutral heteropolysaccharide Uronic acid content: 10.54% Structural feature: Low esterification SEM: Morphology: Irregular fragments Surface characteristics: Smooth surface Structural state: Sharply defined particle boundaries		HPGPC, HPAEC, FT-IR, SEM	Mo et al. (2026)
Fruit hulls	FPW-W	Mw = 156 kDa	Ara: Gal = 1.00: 3.58	FT-IR: 3,385 cm^−1^: O-H stretching; 2,934 cm^−1^: C-H stretching; 1,632 cm^−1^: O-H flexural vibration; 1,154–1,039 cm^−1^: Pyranose ring; 877 cm^−1^: β-D-pyranosidic linkage; 777 and 716 cm^−1^: Furanose ring; 1740 cm^−1^: no absorption (neutral polysaccharide) NMR: Type: AG-II Backbone: 1,3-β-D-Galp Branching: at O-6 position Sugar residues: 1,6-β-D-Galp; terminal-α-L-Araf; 1,3-α-L-Araf; 1,3,6-β-D-Galp; terminal-β-D-Galp; terminal-β-L-Arap Key linkages: 1,3-α-L-Araf (C1) → 1,6-β-D-Galp (C6); Terminal-α-L-Araf (C1) → 1,3-α-L-Araf (C3); Terminal-β-D-Galp (C1) → 1,6-β-D-Galp (C6) SEM: Morphology: Dispersive structure with irregular flaky shapes Structural state: amorphous structure Surface characteristics: Smooth surface at microstructure level Key features: Multi-branched nature; high purity; strong intermolecular interactions TEM: Morphology: Smooth, rounded particles Dispersion: good dispersion in solution Particle diameter: approximately 20 nm		HPGPC, GC-MS, FT-IR, NMR, SEM, TEM	Wu et al. (2020a)
Fruits	FPLP-W	Mw = 34.69 kDa	GalA	FT-IR: 3,424 cm^−1^: O-H stretching; 2,935 cm^−1^: C-H stretching; 1749 cm^−1^: C=O stretching of COOH; 1,623 cm^−1^: O-H flexural vibration; 1,450–1,310 cm^−1^: CH_2_ and C-OH deformations; 1,200–1,020 cm^−1^: C-O stretching vibrations; 960 cm^−1^: C-O(O-CH_2_) stretching NMR: Type: HG-type pectin Backbone: Linear (1,4)-α-D- GalA Branching: 1.30% branched-chain hexenuronic acid Sugar residues: α-GalpA6Me; β-GalpA; T-α-GalpA; 1,4-α-GalpA; 1,4-α-GalpA6Me; α-HexpA; α-HexpA6Me; 1,3,4-α-GalpA6Me		HPGPC, GC-MS, FT-IR, NMR	Wu et al. (2017)
​	DM3	Mw = 42.7 kDa	GalA	Uronic acid: 97.4% DM = 3%, DA = 1.2% Shake-flask method: logP = −1.253 DLS: Particle size 316.2 nm	FT-IR: 3,424 cm^−1^: O-H stretching 2,935 cm^−1^: C-H stretching (CH_2_ and CH_3_) ∼1740 cm^−1^: C=O stretching of esterified carbonyl 1,630–1,600 cm^−1^: COO^−^ stretching 1,435 cm^−1^: asymmetric stretching of esterified -CH_3_ 1,365 cm^−1^: Symmetrical stretching of esterified -CH_3_ FT-IR Trends As DM increases: 1740 cm^−1^ (esterified C=O): Absorption intensity gradually increases 1,435 cm^−1^ and 1,365 cm^−1^ (esterified -CH_3_): Absorption intensity gradually increases 1,630–1,600 cm^−1^ (COO^−^): Absorption intensity gradually decreases Overall spectrum: Spectral shape changes, with peak positions shifting to higher wavenumbers	HPGPC, FT-IR, NMR, DLS, SEM, m-Hydroxybiphenyl method, Phenol-sulfuric acid method, Shake-flask method	Chen et al. (2022)
	DM16	Mw = 46.5 kDa		Uronic acid: 97.2% DM = 16%, DA = 1.2% Shake-flask method: logP = −0.789 DLS: Particle size 358.5 nm			
	FPLP/DM25	Mw = 50.5 kDa		Uronic acid: 98.7% DM = 25%, DA = 2.0% Shake-flask method: logP = −0.646 DLS: Particle size 441.5 nm			
	DM34	Mw = 51.5 kDa		Uronic acid: 98.3% DM = 34%, DA = 2.0% Shake-flask method: logP = −0.540 DLS: Particle size 266.4 nm			
	DM45	Mw = 52.6 kDa		Uronic acid: 97.0% DM = 45%, DA = 2.0% Shake-flask method: logP = −0.449 DLS: Particle size 253.4 nm			
​	​	​	​	​	NMR: Type: HG-type pectin Backbone: Linear (1,4)-α-D- GalA NMR Trends As DM increases: ^1^H NMR: Methyl ester signal (∼3.80 ppm) gradually increases (relative to H-4 signal at 4.41 ppm) ^13^C NMR: Free carboxyl signal (175.56 ppm) gradually decreases Esterified carboxyl signal (171.53 ppm) gradually increases Methyl ester carbon signal (53.50 ppm) gradually increases Carboxyl signal shifts from 175.56 ppm to 171.53 ppm (conversion of -COOH to -COOCH_3_)	​	​
	DM54	Mw = 67.3 kDa		Uronic acid: 99.8% DM = 54%, DA = 2.0% Shake-flask method: logP = −0.321 DLS: Particle size 239.1 nm			
	DM63	Mw = 48.4 kDa		Uronic acid: 98.9% DM = 63%, DA = 1.5% Shake-flask method: logP = −0.571 DLS: Particle size 246.6 nm			
	DM76	Mw = 45.7 kDa		Uronic acid: 97.0% DM = 76%, DA = 1.2% Shake-flask method: logP = −0.508 DLS: Particle size 288.0 nm			
	DM84	Mw = 43.8 kDa		Uronic acid: 97.0% DM = 84%, DA = 1.0% Shake-flask method: logP = −0.577 DLS: Particle size 299.2 nm			
	DM94	Mw = 38.0 kDa		Uronic acid: 97.0% DM = 94%, DA = 0.8% Shake-flask method: logP = −0.569 DLS: Particle size 298.7 nm			
	DM3	Mw = 42.7 kDa	GalA	Uronic acid: 97.4%, DM = 3%, DA = 1%		HPLC, NMR, m-Hydroxydiphenyl method	Chen et al. (2024)
	FPLP/DM25/DA2	Mw = 50.5 kDa		Uronic acid: 98.7%, DM = 25%, DA = 2%			
	DM54	Mw = 67.3 kDa		Uronic acid: 99.8%, DM = 54%, DA = 2%			
	DM94	Mw = 38.0 kDa		Uronic acid: 97.0%, DM = 94%, DA = 0.8%			
	DA23	Mw = 23.1 kDa		Uronic acid: 97.2%, DM = 25%, DA = 23%			
​	FPP-Y	Mw = 645.776 kDa	Fuc: Rha: Ara: Gal: Glc: Xyl: Man: GalA: GlcA = 0.33: 0.57: 1.46: 3.69: 1.00: 0.34: 0.06: 2.69: 0.17	FT-IR: 3,400 cm^−1^: O-H stretching vibration, 2,940 cm^−1^: C-H stretching vibration, 1,610 cm^−1^: Bound water, 1,410 cm^−1^: Carboxyl group asymmetric stretching, 1,000–1,200 cm^−1^: C-O-C and C-O-H bonds, 1746 cm^−1^ (FPP-Y): C=O stretching Chemical composition: FPP-Y: Neutral sugars: 55.30% ± 6.66%; uronic acid: 45.41% ± 0.52%; protein: 10.45% ± 0.36% FPW-Y: Neutral sugars: 39.1% ± 1.14%; uronic acid: 34.6% ± 1.47%; protein: 15.69% ± 0.25% Hydrodynamic Diameter: FPP-Y: 385.6 nm; FPW-Y: 274.0 nm Zeta potential: FPP-Y: −26.2 mV; FPW-Y: −19.4 mV TGA: FPW-Y (56.25% weight loss) > FPP-Y (69.5% weight loss) SEM: FPP-Y: smooth, flat surfaces; FPW-Y: rough, fragmented morphology		HPLC, Bradford assay, Carbazole-Sulfuric acid method, Phenol-Sulfuric acid method, FT-IR, IC, TGA, SEM, DLS, Zeta potential	Ye et al. (2024), Ye et al. (2025)
	FPW-Y	N/A	Fuc: Rha: Ara: Gal: Glc: GalA: GlcA = 0.75: 3.31: 16.31: 13.08: 1.00: 21.60: 1.73				
	FPP-M	Mw = 966.70 kDa	Fuc: Rha: Ara: Gal: Glc: Xyl: Man: GalA: GlcA = 0.30: 0.58: 1.27: 3.54: 1.00: 0.38: 0.06: 2.33: 0.12	FT-IR: 3,410 cm^−1^: O–H stretching; 2,931 cm^−1^: C–H stretching; 1743 cm^−1^: Esterified carboxyl group (C=O) 1,609 cm^−1^: COO− asymmetric stretching; 1,102 and 1,019 cm^−1^: Pyranose ring vibrations Type: acidic pectic polysaccharide Uronic acid content: 44.99% Structural feature: GalA-rich pectin SEM: Morphology: Dense lamellar structure Surface characteristics: Relatively smooth surface Key features: Slight curling and indentation		HPGPC, HPAEC, FT-IR, SEM	Mo et al. (2026)
Seeds	FPL pectin	Mw = 5.14 × 10^4^ kDa Mn = 1.94 × 10^3^ kDa	N/A	Chemical composition: GalA content: 88.96%		N/A	Luo et al. (2024)
	CFSP	160.8 kDa	N/A	N/A		N/A	Liu et al. (2021)
	Pectin-C	N/A	GalA: Rha: Ara: Man = 87.03: 0.74: 1.77:0.81	DM = 20.63 ± 0.12%		N/A	Chen et al. (2021)
	WEP	Mv = 359.2 kDa	N/A	FT-IR: 3,440 cm^−1^: O-H stretching vibration, 2,929 cm^−1^: C-H stretching, 1744 cm^−1^: C=O stretching (methyl esterified carboxyl), 1,630 cm^−1^: O C-O vibration (free carboxyl), 1,106 cm^−1^: Backbone vibration, 1,012 cm^−1^: C-C vibration Chemical composition: GalA content: WEP (87.73%), ChEP (85.47%), HEP (77.92%) Ash content: HEP (2.33%), WEP (1.17%), ChEP (0.22%) Protein content: HEP (3.23%), ChEP (0.28%), WEP (0.17%)		FT-IR, GC, Ubbelohde Viscometer, ICP-OES, Inverted Microscope	Liang et al. (2012)
	ChEP	Mv = 251.1 kDa	N/A				
	HEP	Mv = 78.6 kDa	N/A				
​	WEP-0.3	N/A	N/A	FT-IR: 1742 cm^−1^: Methyl-esterified carboxyl group, 1,627 cm^−1^: Free carboxyl group DM = 10.46% XRD: Amorphous structure Type: Low-methoxyl pectic polysaccharide ESEM: Porous sponge-like morphology Mineral analysis: Ca^2+^ content = 3.26 mg/g		FT-IR, XRD, ESEM, ICP-AES	Wang et al. (2019)
	WEP-0.4			FT-IR: 1742 cm^−1^: Methyl-esterified carboxyl group, 1,627 cm^−1^: Free carboxyl group DM = 6.67% XRD: Crystalline structure with distinct crystal peaks Type: Low-methoxyl pectic polysaccharide ESEM: Dense compact rod-like morphology Entangled aggregate structure Mineral analysis: Ca^2+^ content = 6.84 mg/g			
Stems	FPSP	Mw = 201.58 kDa	Fuc: Rha: Ara: Gal: Glc: Xyl: Man: GalA: GlcA = 0.25: 0.33: 0.07: 1.09: 1.00: 0.10: 0.18: 0.10: 0.07	FT-IR: 3,366 cm^−1^: O–H stretching; 2,930 cm^−1^: C–H stretching; 1,605 cm^−1^: COO− asymmetric stretching; 1,032 cm^−1^: neutral sugar-associated vibration; 1740 cm^−1^: no obvious absorption peak Type: neutral heteropolysaccharide Uronic acid content: 16.94% Structural feature: Low esterification SEM: Morphology: Irregular fragments Surface characteristics: Smooth edges Structural state: Compact structure		HPGPC, HPAEC, FT-IR, SEM	Mo et al. (2026)

N/A: information was not available.

### 4.1 Molecular weight

The Mw distribution of creeping fig polysaccharide is wide, and there are significant differences due to different sources and preparation methods. Among them, the Mw of creeping fig syconium polysaccharides was generally high, with FPP-M being the most prominent, with a Mw of 966.70 kDa, which was a typical high Mw acidic polysaccharide (Mo et al., 2026). The Mw of FPLP-W further purified by ion exchange and gel filtration was only 34.69 kDa, and the difference between the two was very large, indicating that the purification and classification would significantly reduce the Mw of polysaccharides (Wu et al., 2017). The Mw of stem and leaf polysaccharides (FPSP and FPLP-M) were similar, which were 201.58 kDa and 202.65 kDa, respectively (Mo et al., 2026). The FPW-W was 156 kDa, which was at a medium level. The Mw of achene polysaccharide changed significantly with different extraction methods. The viscosity-average Mw of WEP extracted by water was 359.2 kDa, ChEP extracted by chelating agent was 251.1 kDa, and HEP extracted by acid was only 78.6 kDa, indicating that acidic conditions would lead to a certain degree of degradation of pectin molecular chains (Liang et al., 2012). It can be seen that the source site, extraction method and purification degree jointly determine the Mw of creeping fig polysaccharides. It should be noted that the Mw of FPL pectin was reported as 5.14 × 10^4^ kDa. This value is markedly higher than those of most reported creeping fig polysaccharides and should therefore be interpreted cautiously as an apparent Mw, which may be influenced by the large hydrodynamic volume, chain aggregation or network association of pectic polysaccharides.

### 4.2 Monosaccharide composition

The monosaccharide composition of creeping fig polysaccharide varies from source to source, showing obvious tissue specificity. In general, creeping fig polysaccharides were mainly composed of GalA, galactose (Gal), arabinose (Ara), glucose (Glc), rhamnose (Rha), fucose (Fuc), xylose (Xyl) and mannose (Man), but the types and proportions of monosaccharides in polysaccharides from different parts were significantly different. GalA was the main monosaccharide of polysaccharides from syconium and achenes, showing typical pectin characteristics. Among them, the GalA content of achene pectin was generally higher, reaching 88.96% in FPL pectin and 87.73% in WEP (Liang et al., 2012; Wang et al., 2019). The contents of GalA in the syconium polysaccharides FPP-M and FPP-Y were 44.99% and 45.41%, respectively (Mo et al., 2026; Ye et al., 2025). The higher GalA content indicates that these polysaccharides have a large proportion of pectin structures, which is also closely related to their acidic characteristics and gel formation ability. In contrast, polysaccharides from stems and leaves were mainly composed of Gal and Glc, and contained a small amount of neutral monosaccharides such as Ara, Rha, Fuc, Xyl and Man. The content of GalA was relatively low (such as FPLP-M was only 10.54%, FPSP was 16.94%) (Mo et al., 2026). This composition indicates that the proportion of pectin structures in stem and leaf polysaccharides is low, while neutral heteropolysaccharides dominate. At the same time, FPW-W is composed of Ara and Gal (Ara: Gal = 1.00: 3.58), which is a heteropolysaccharide rich in Gal, and its composition characteristics are consistent with the structure of AG-II obtained by subsequent identification (Wu et al., 2020a).

It is worth noting that GalA is mainly enriched in syconium and achene polysaccharides, while the content of GalA in stems, leaves and fruit hull polysaccharides is significantly reduced. On the contrary, the proportion of neutral sugars such as Gal and Ara in stems, leaves and fruit hulls was relatively high. This reflects the difference of polysaccharide structure types in different tissues of creeping fig. In addition to the source parts, the differences in extraction methods, purification processes and analytical methods may also affect the determination results of monosaccharide composition and molar ratio (Zhao et al., 2023).

### 4.3 Chemical structures

The chemical structures of creeping fig polysaccharides showed diversity, mainly including two structural types of pectin polysaccharides and neutral heteropolysaccharides. Pectin polysaccharides mainly exist in the achenes and syconium of creeping fig. FPLP-W, a syconium polysaccharide, is a homogalacturonan (HG) type pectin (Wu et al., 2017). The main chain is composed of (1→4)-α-D-GalA and contains a small amount of branched chains. Achene pectins are mostly low-methoxyl pectins. For example, the degree of esterification of WEP-0.3 and WEP-0.4 were 10.46% and 6.67%, respectively, which were lower than 50%. These low methoxyl pectins can form gels through calcium ion crosslinking, which is the structural basis of the gel properties of creeping fig achenes. FT-IR analysis showed that the characteristic absorption of methyl esterified carbonyl group (C=O) appeared near 1740–1745 cm^−1^, and the absorption of free carboxyl group (COO^−^) appeared near 1,600–1,630 cm^−1^, confirming its pectin properties. Neutral heteropolysaccharide mainly exists in the stems, leaves and fruit hulls of creeping fig. Both stem polysaccharide FPSP and leaf polysaccharide FPLP-M were neutral heteropolysaccharides with low degree of esterification and low content of uronic acid. FT-IR had no obvious absorption peak near 1740 cm^−1^, which further confirmed the characteristics of neutral polysaccharides. FPW-W was a type II arabinogalactan (AG-II) with a main chain of (1→3)-β-D-Gal, and branched at the O-6 position, connecting Ara and Gal residues, showing highly branched structures (Wu et al., 2020a).

In summary, creeping fig polysaccharides showed significant differences in Mw, monosaccharide composition and chemical structures. Achene and syconium polysaccharides are mostly acidic pectic polysaccharides rich in GalA, while stem, leaf and fruit hull polysaccharides are mostly neutral heteropolysaccharides. However, the current research on the structures of creeping fig polysaccharides is still uneven. Some polysaccharides still lack fine glycosidic bond connections and high-level conformational information. In the future, methylation analysis, NMR, mass spectrometry and other methods are still needed to further clarify their fine structures (Zhang et al., 2017).

## 5 Biological activities of creeping fig polysaccharides

**FIGURE 4:**
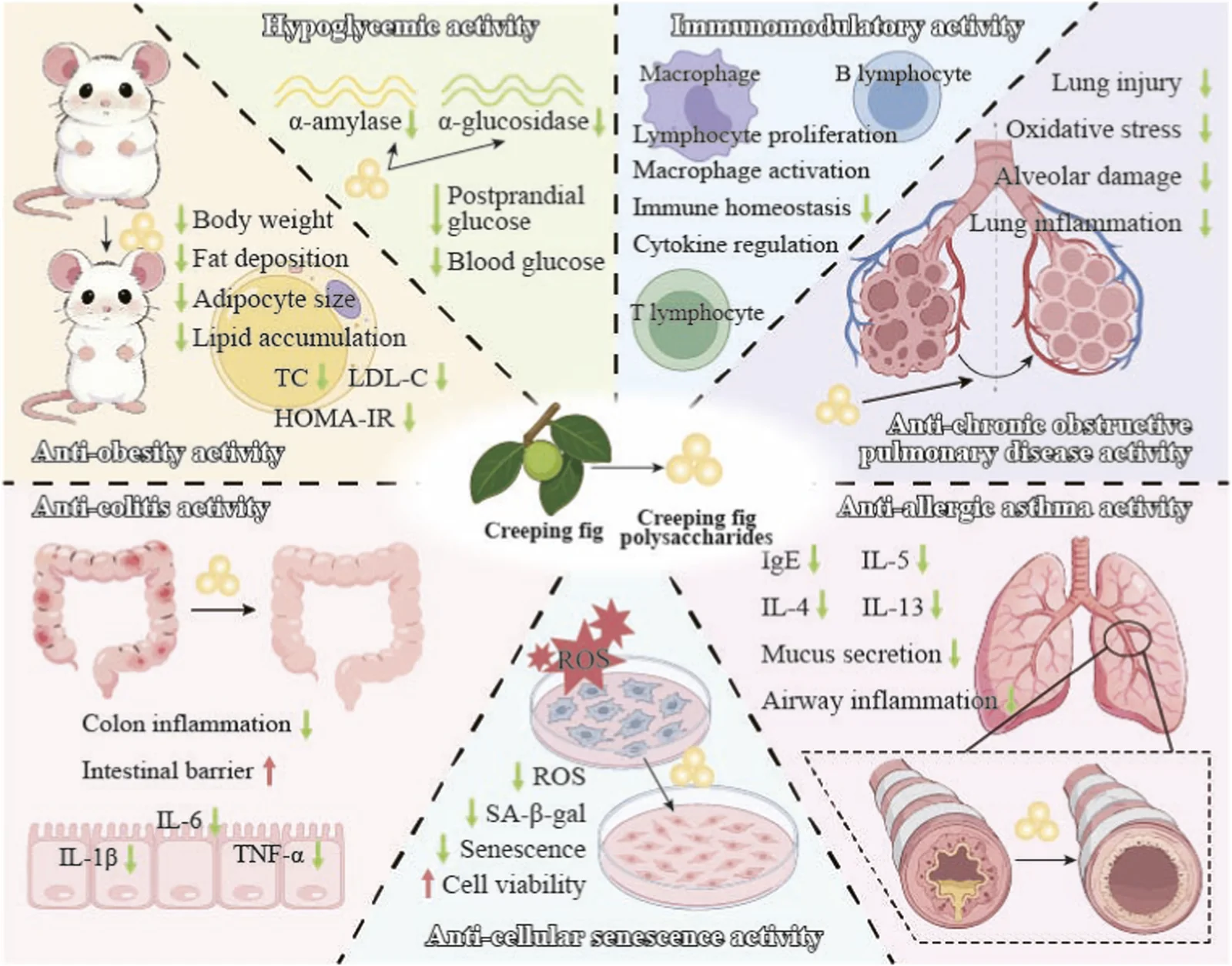
The biological activities of creeping fig polysaccharides.

Creeping fig polysaccharide is an important material basis for creeping fig to exert its edible and medicinal value. At present, researchers have extensively explored the biological activities of creeping fig polysaccharide through *in vivo* and *in vitro* experiments, and confirmed that it has anti-colitis, anti-obesity, anti-allergic asthma, anti-chronic obstructive pulmonary disease, hypoglycemic, anti-cellular senescence and immune regulation and other activities (Figure 4). Table 3 summarizes the biological activities and action mechanisms of creeping fig polysaccharides.

**TABLE 3:** Biological activities of creeping fig polysaccharides.

Biological activities	Polysaccharide name	Evidence level	Indicated concentrations	Models/test system	Action or mechanism	Ref
Anti-colitis activity	DM3	*In vivo*	300 mg kg^−1^	Female C57BL/6J mice	TNF-α **↓**, IL-1β **↓**, IL-6 **↓**, IL-17 **↓** by 58.8%, 62.6%, 74.7%, and 78.0%, myeloperoxidase (MPO) activity **↓**, serum LPS ↓, serum DAO ↓, *Lactobacillus* **↑,** Lachnospiraceae*_NK4A136_*group **↑,** Prevotellaceae **↓**	Chen et al. (2024)
	FPLP/DM25/DA2				TNF-α ↓, IL-1β ↓, IL-6 ↓, IL-17 ↓, serum LPS ↓, serum DAO ↓, *Candidatus_Saccharimonas* **↑**↑, *Escherichia-Shigella* ↓	
	DM54				TNF-α ↓, IL-6 ↓, MPO activity **↓**, serum LPS ↓, colon length ↑, Firmicutes/Bacteroidota ratio ↑, Lachnospiraceae*_NK4A136_group* ↑, *Escherichia-Shigella* ↓	
	DM94				Serum LPS ↓↓, serum DAO ↓↓ by 57.5%, *Bifidobacterium* ↑↑, *Mucispirillum* ±	
	DA23				TNF-α ↓, IL-6 ↓, IL-17 ↓, serum DAO ↓, Lachnospiraceae*_NK4A136_group* ↑, *Mucispirillum* ↑, *Escherichia-Shigella* ↑	
Anti-obesity activity	FPLP-W	*In vivo*	200 mg kg^−1^	Male C57BL/6 mice	Firmicutes/Bacteroidetes ratio ↓, *Akkermansia* ↑, *Blautia* ↓, myristoleic acid ↑, pentadecanoic acid ↑, serum TC ↓, serum LDL-C ↓, HOMA-IR ↓, serum Leptin ↓, serum adiponectin ↑, TNF-α ↓, IL-6 ↓, MCP-1 ↓	Wu et al. (2020b)
Anti-allergic asthma activity	FPP-Y	*In vivo*	100, 200 mg kg^−1^	Male ICR mice	IL-4 ↓, IL-5 ↓, IL-13 ↓, IFN-γ ↑, MMP-9, TIMP-1, α-SMA ↓, SCFAs ↑, gut microbiota alpha diversity (Chao1, Faith-pd, observed-species, Shannon) ↑	Ye et al. (2024)
	FPW-Y					
	FPP-Y	*In vivo*	100, 200 mg kg^−1^	Male ICR mice	MAPK/NF-κB signaling pathway, p-p65/p65 ↑, p-IκBα/IκBα ↑, p-JNK/JNK ↑, p-ERK/ERK ↓, p-p38/p38 ↓, IL-5 in BALF ↓, IL-13 in BALF ↓, SOD activity ↑, MDA level ↓, TNF-α level in colon ↓	Ye et al. (2025)
	FPW-Y					
Anti-chronic obstructive pulmonary disease activity	FPP-C	*In vivo*	50 mg kg^−1^	Cigarette smoke-induced COPD mice	IL-1β ↓, IL-6 ↓, TNF-α ↓, Alveolar deformation ↓, airway wall thickening ↓, inflammatory cell infiltration ↓, collagen deposition ↓, goblet cell hyperplasia ↓, mucus secretion ↓, gut microbiota α-diversity (Chao1/Faith_pd/Shannon/Pielou_e/Observed_species/Simpson) ↑, Firmicutes ↑, Bacteroidetes ↑, Firmicutes/Bacteroidetes ratio ↓, Clostridiaceae ↑, Staphylococcaceae ↑, [Prevotella] ↑, Oscillospira ↑, Ruminococcus ↑, Helicobacteraceae ↓, Campylobacteraceae ↓, *Helicobacter* ↓, Allobaculum ↓, Parabacteroides ↓, total SCFAs ↑ by 41.90%, butyrate ↑ by 23.41%, propionate ↑ by 45.45%, Butanoate biosynthesis ↑, carbohydrate degradation ↑, Amino acid/cofactor metabolism ↑	Chen et al. (2026)
	FPLP-C					
Hypoglycemic activity	FPLP	*In vivo*	100, 200 mg kg^−1^	C57BL/KsJ db/db mice	AMPK/GSK-3β/GS pathway, phospho-AMPKα-Thr172 ↑, p-GSK-3β-Ser9 ↑, p-GS-Ser641 ↓, IRS-1/PI3K/Akt/GSK-3β/GS pathway, p-IRS-1-Ser1101 ↓, PI3K ↑, p-Akt-Ser473 ↑, p-GSK-3β-Ser9 ↑, p-GS-Ser641 ↓, fasting blood glucose ↓, serum insulin ↓, HOMA-IR ↓, hepatic glycogen content ↑, GK expression ↑, G6Pase expression ↓, PEPCK expression ↓	Wu et al. (2017)
	DM3	*In vitro*	0, 200, 400, 500, 600, 800, 1,000 μg mL^−1^	IR-HepG2 cell	DM regulates cellular uptake by modulating macropinocytosis and caveolae-mediated endocytosis, thereby affecting intracellular drug concentration. As DM increases (0→100), the hypoglycemic activity and cellular uptake of HG follow an inverted U-shaped trend, peaking at DM54	Chen et al. (2022)
	DM16					
	FPLP/DM25					
	DM34					
	DM45					
	DM54					
	DM63					
	DM76					
	DM84					
	DM94					
Anti-cellular senescence activity	FPP-M	*In vitro*	25, 50, 100 μg mL^−1^	CSE-induced Beas-2b cells	SA-β-gal activity ↓, G0/G1 arrest ↓, mitochondrial membrane potential ↑, Nrf2 ↑, 133.2% at 50 μg mL^−1^, Keap1 modulation, oxidative stress ↓	Mo et al. (2026)
	FPSP				SA-β-gal activity ↓, G0/G1 arrest ↓, mitochondrial membrane potential ↑, Nrf2 signaling modulation, 51.5% at 100 μg mL^−1^	
	FPLP-M				SA-β-gal activity ↓, mitochondrial depolarization ↓, G1/S transition restored, Nrf2 pathway activation, 108.0% at 100 μg mL^−1^, oxidative stress suppression	
Immunomodulatory activity	FPW-W	*In vitro*	25, 50, 100, 200, 400 μg mL^−1^	Murine RAW 264.7 macrophage cell line	Phagocytic activity ↑, NO ↑, maximum = 1.97-fold at 200 μg mL^−1^, TNF-α↑, IL-6 ↑, IL-1β ↑	Wu et al. (2020a)

↑: Indicates upregulation, increase, or a promoting effect.

↑↑: Indicates a strong upregulation, increase, or promoting effect.

↓: Indicates downregulation, decrease, or an inhibitory effect.

↓↓: Indicates a strong downregulation, decrease, or inhibitory effect.

±: Indicates a modulating/normalizing effect with a non-uniform direction.

### 5.1 Anti-colitis activity

Colitis is characterized by disruption of the mucosal barrier, immune imbalance, and dysbiosis (Qian et al., 2025). The dextran sulfate sodium (DSS)-induced model reliably replicates the typical inflammatory phenotype of colitis, making it a classic experimental system for evaluating intervention strategies (Vlantis et al., 2016; Li et al., 2024). Natural polysaccharides exhibit unique advantages in the intervention of colitis due to their multitarget anti-inflammatory activities (Guo et al., 2022; Ma et al., 2023).

**FIGURE 5:**
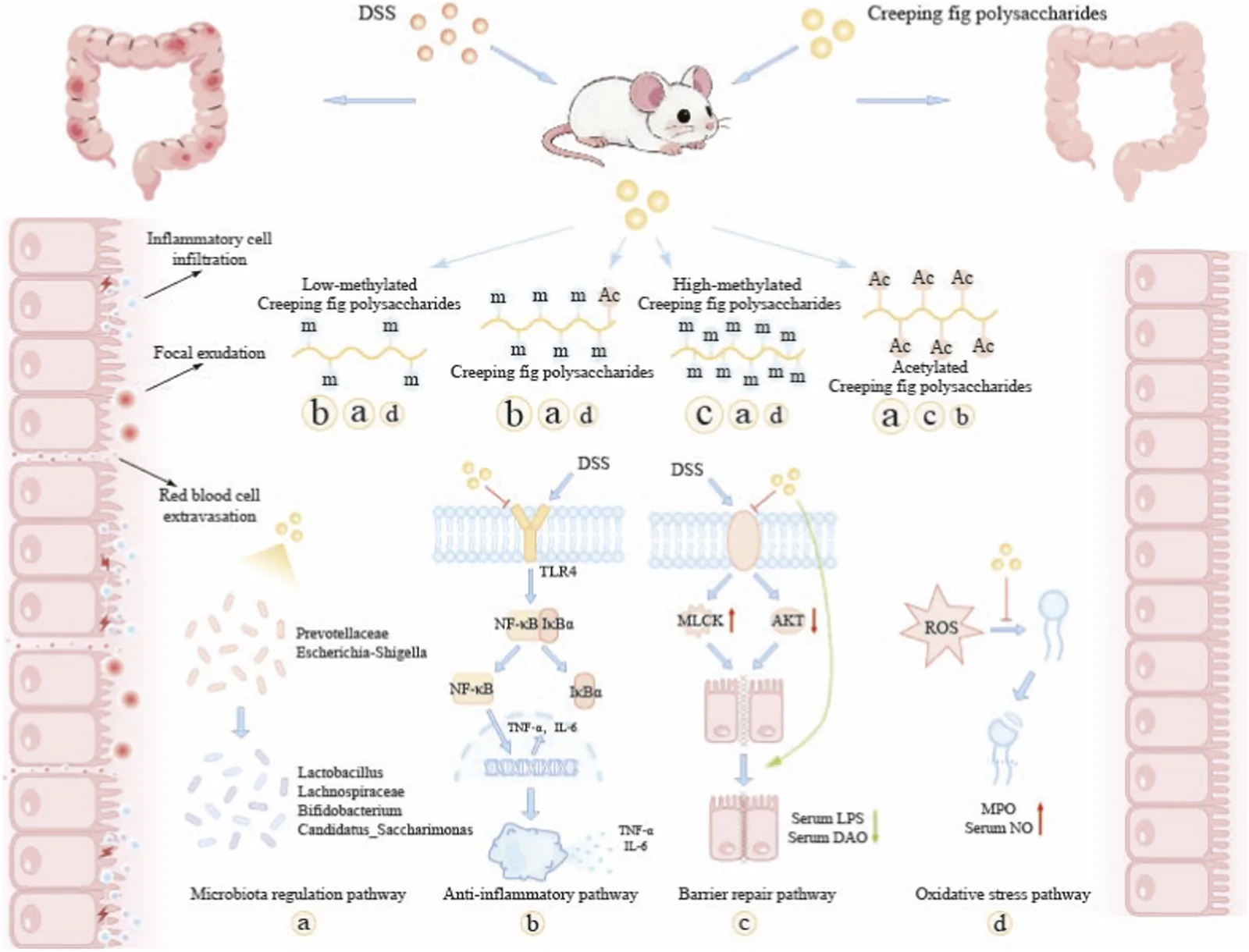
Mechanism of creeping fig polysaccharides in alleviating colitis.

Chen et al. (2024) evaluated the anti-colitis activity of homogalacturonan (HG)-type pectin from creeping fig in a 3% DSS-induced colitis mouse model (Figure 5). In this study, mice were treated with 5-aminosalicylic acid (5-ASA, 100 mg kg^−1^·day^−1^) as the positive control or HG derivatives with different degrees of methylesterification (DM) and acetylation (DA) at 300 mg kg^−1^·day^−1^ for 7 days. The tested samples included DM3, DM25/DA2, DM54, DM94, and DA23. This experimental design allowed comparison of the effects of methylesterification and acetylation on the anti-colitis activity of HG-type pectin. The results showed that HG derivatives alleviated DSS-induced colitis-related symptoms to different degrees, as reflected by reduced body weight loss, lower disease activity index scores, alleviated colon shortening, decreased spleen index, and improved colonic histopathological damage. Among the samples with different DM values, low-DM HG, especially DM3 and DM25, showed stronger effects on body weight maintenance, spleen index reduction, and inflammatory cytokine inhibition, whereas high-DM HG, especially DM94, appeared more effective in improving intestinal barrier-related indicators. For example, DM3 reduced the levels of TNF-α, IL-1β, IL-6, and IL-17 in colonic tissues by 58.8%, 62.6%, 74.7%, and 78.0%, respectively. In contrast, DM94 showed a more pronounced effect on reducing serum levels of lipopolysaccharide (LPS) and diamine oxidase (DAO), which are commonly used as indicators of intestinal barrier damage. In addition, all HG derivatives reduced oxidative stress-related indicators, such as nitric oxide levels, although DM and DA had relatively limited influence on this effect. Gut microbiota modulation was also observed after HG intervention. Low-DM HG tended to increase the abundance of beneficial bacteria such as *Lactobacillus* and Lachnospiraceae NK4A136 group, whereas high-DM HG showed different regulatory tendencies toward bacteria such as Bifidobacterium, Mucispirillum, and *Bacteroides*. Acetylated HG, such as DA23, showed reduced water solubility due to the introduction of hydrophobic acetyl groups, and its inhibitory effect on several inflammatory cytokines was weaker than that of low-DM HG. However, DA23 displayed a distinct microbiota-regulating profile, including effects on Lachnospiraceae NK4A136 group, Mucispirillum, and Escherichia-Shigella. These findings suggest that DM and DA may influence the anti-colitis activity of HG by affecting inflammatory responses, intestinal barrier indicators, oxidative stress, and gut microbiota composition.

Overall, current evidence indicates that HG-type pectin from creeping fig can alleviate colitis-related pathological changes in a DSS-induced mouse model. The experimentally observed outcomes include reduced inflammatory cytokine levels, improved intestinal barrier-related markers, decreased oxidative stress indicators, and altered gut microbiota composition. However, the causal relationships among structural modification, gut microbiota changes, barrier repair, and inflammatory regulation remain incompletely verified. In addition, the current evidence is limited to an acute DSS-induced mouse model, and pharmacokinetic behavior, dose-response relationships, long-term safety, and clinical efficacy have not been established. Future studies should further clarify the key signaling pathways, verify microbiota-related causality, and conduct pharmacokinetic and toxicological evaluations.

### 5.2 Anti-obesity activity

Obesity is a chronic metabolic disorder characterized by excessive adipose tissue accumulation and is closely associated with insulin resistance, dyslipidemia, and chronic low-grade inflammation (Vecchié et al., 2018; Marcelin et al., 2019). Increasing evidence suggests that natural polysaccharides may alleviate obesity-related metabolic disorders through the regulation of lipid metabolism and inflammatory responses (He et al., 2022; Liu et al., 2022). Wu et al. (2020b) investigated the anti-obesity activity of FPLP-W, a polysaccharide isolated from creeping fig syconium, in high-fat diet-induced obese mice. In this study, FPLP-W was orally administered at 200 mg kg^−1^·day^−1^ for 17 weeks. Long-term administration of FPLP-W significantly reduced body weight gain, adipose tissue accumulation, and adipocyte hypertrophy. Histological observations further showed that FPLP-W effectively alleviated adipocyte enlargement caused by a high-fat diet, indicating an inhibitory effect on excessive lipid deposition. In addition, FPLP-W improved glucose tolerance and insulin sensitivity, suggesting a beneficial role in maintaining systemic metabolic homeostasis. FPLP-W also exerted favorable effects on lipid metabolism. Treatment significantly decreased serum total cholesterol (TC) and low-density lipoprotein cholesterol (LDL-C) levels, while reducing obesity-associated inflammatory responses and increasing adiponectin expression. As adiponectin is an important adipokine involved in glucose and lipid metabolism, its elevation may contribute to improved insulin sensitivity and metabolic regulation. Further analysis showed that FPLP-W also modulated gut microbiota and fecal metabolites, including a decreased Firmicutes/Bacteroidetes ratio, enrichment of Akkermansia, reduction of Blautia, and changes in metabolites such as myristoleic acid and pentadecanoic acid. These findings suggest that FPLP-W may ameliorate obesity-related metabolic dysfunction through the coordinated regulation of lipid metabolism, inflammation, gut microbiota, and related metabolites.

Overall, current evidence indicates that creeping fig polysaccharides possess considerable anti-obesity potential and may contribute to the improvement of obesity-related metabolic disorders. By simultaneously regulating body weight gain, lipid accumulation, glucose homeostasis, and inflammatory status, FPLP-W exhibits a multi-target metabolic regulatory effect. However, the available evidence is still derived from a high-fat diet-induced mouse model, and the relationships among gut microbiota changes, fecal metabolites, and metabolic improvement are mainly based on correlation analysis. Dose-response relationships, causal mechanisms, long-term safety, and clinical efficacy remain to be further verified.

### 5.3 Anti-allergic asthma activity

**FIGURE 6:**
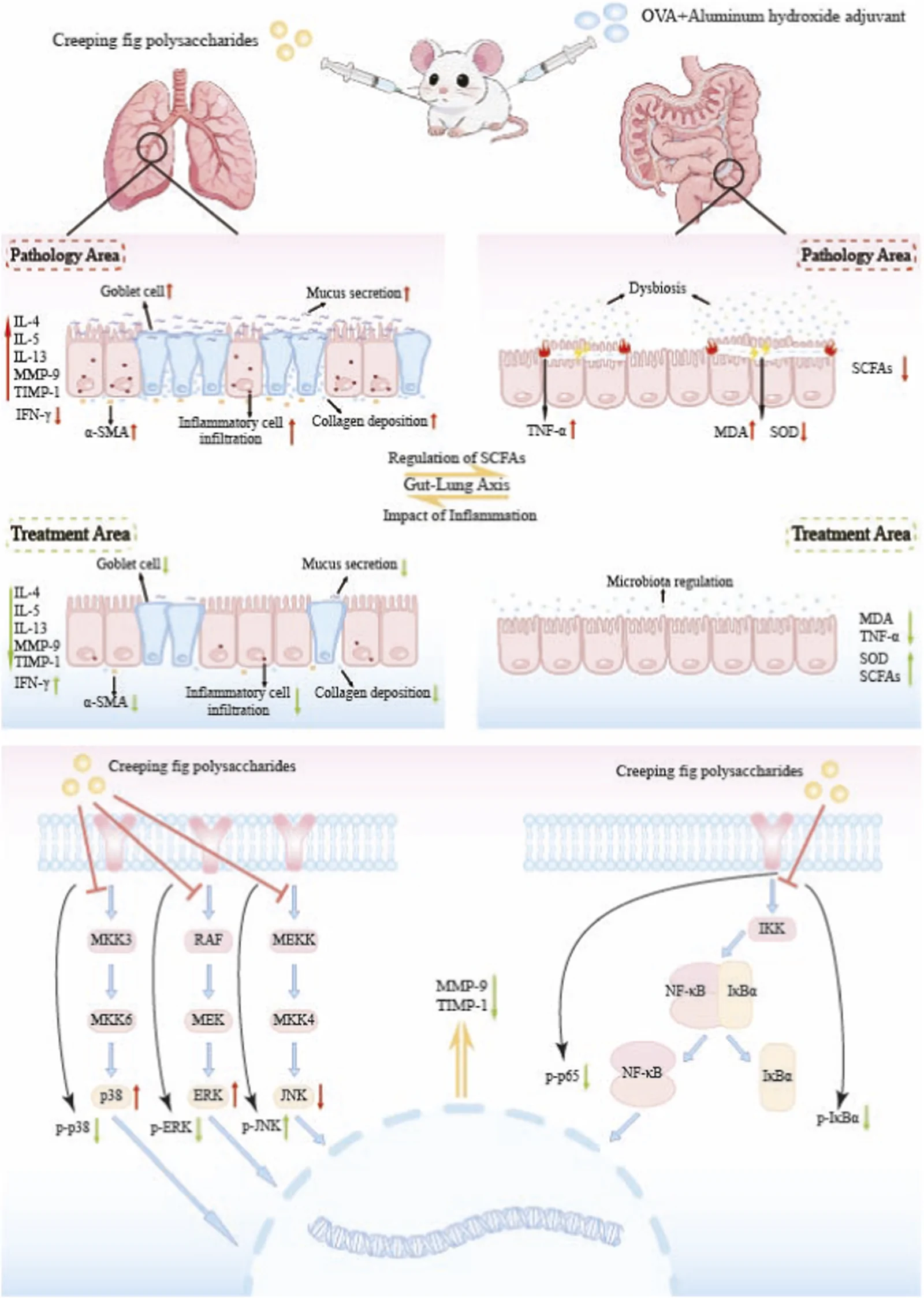
Mechanism of creeping fig polysaccharides in alleviating allergic asthma.

Allergic asthma is triggered by exogenous allergens and is characterized by persistent airway inflammation and immune imbalance (Schatz and Rosenwasser, 2014). Although existing glucocorticoid therapy can relieve symptoms, it has obvious long-term side effects and is difficult to cure (Lin and Casale, 2002; Oray et al., 2016). Therefore, natural intervention strategies with multi-target regulatory potential have attracted increasing attention. FPP-Y and its water extract FPW-Y from creeping fig showed significant alleviating effects on ovalbumin (OVA)-induced asthma model in mice (Ye et al., 2024; Ye et al., 2025) (Figure 6). In these studies, male ICR mice were sensitized and challenged with OVA, and FPP-Y or FPW-Y was administered by gavage at 100 and 200 mg kg^−1^·day^−1^, with dexamethasone (Dex, 9.75 mg kg^−1^·day^−1^) as the positive control. Both of them can significantly reduce airway inflammatory infiltration, goblet cell proliferation and mucus secretion, and inhibit the abnormal expression of airway remodeling-related proteins (such as MMP-9, TIMP-1 and α-SMA), suggesting that they have potential value in delaying airway structural remodeling. At the level of immune regulation, FPP-Y and FPW-Y can effectively correct Th1/Th2 immune imbalance, reduce the levels of Th2 cytokines such as IL-4, IL-5 and IL-13, and partially restore the expression of IFN-γ, thereby alleviating allergic inflammation. Further molecular analysis showed that both of them could inhibit the abnormal activation of MAPK/NF-κB signaling pathway and reduce the release of pro-inflammatory mediators, suggesting that their anti-inflammatory effects may be associated with the regulation of key signaling nodes rather than the inhibition of a single inflammatory factor. It is worth noting that this lung improvement does not occur in isolation. FPP-Y and FPW-Y simultaneously improved the oxidative stress state of the colon (increased superoxide dismutase (SOD) activity and decreased malondialdehyde (MDA) level) and repaired the morphological damage of intestinal tissue. At the same time, they increased the α diversity of intestinal flora, promoted the recovery of some beneficial flora of Bacteroidetes and Firmicutes, and increased the production of short-chain fatty acids (SCFAs). As important immunomodulatory molecules, SCFAs may participate in pulmonary inflammation regulation through the microbiota-metabolite-host axis. These findings support the possibility that creeping fig polysaccharides may participate in systemic immune regulation through a lung-gut interaction pattern.

However, it should be noted that the current evidence on the relationship between microbiota changes, SCFA production, and the improvement of lung inflammation is still mainly based on correlation analysis, and causal verification experiments such as fecal microbiota transplantation, antibiotic depletion, or metabolite intervention are still lacking. In addition, the effect of the crude extract FPW-Y was better than that of the purified polysaccharide FPP-Y on multiple endpoint indicators. This finding suggests that, in addition to polysaccharides, other bioactive metabolites in the water extract of creeping fig syconium (such as flavonoids and organic acids) may act synergistically with polysaccharides. Their specific contribution ratios and molecular interaction mechanisms remain to be elucidated. Future studies should further clarify the active material basis, receptor recognition mechanism, downstream signaling pathways and causal contribution of gut microbiota.

### 5.4 Anti-chronic obstructive pulmonary disease activity

Chronic obstructive pulmonary disease (COPD) is characterized by persistent airflow limitation and pulmonary inflammatory response (Lareau et al., 2019; Ritchie and Wedzicha, 2020). It has different pathological mechanisms from allergic asthma, but it also involves the interaction between inflammatory imbalance and lung-gut axis (Wang et al., 2023; Xu et al., 2024). Recent studies have revealed a new mechanism by which creeping fig polysaccharides alleviate COPD through regulation of the gut microbiota-SCFAs-lung signaling pathway. In a cigarette smoke-induced COPD mouse model, creeping fig polysaccharide (FP-P) and its water extract (FP-E) significantly attenuated lung pathological damage, reduced inflammatory factor levels, and restored gut microbiota α-diversity (Chen et al., 2026). In this study, mice were exposed to CS for 6 weeks, and FP-P or FP-E was administered by gavage during weeks 3–6 at 50 mg kg^−1^, with theophylline at 2.0 mg kg^−1^·day^−1^ as the positive control. At the level of microbiota composition, FP-P selectively enriched SCFAs-producing Clostridiaceae and Staphylococcaceae, while inhibiting pro-inflammatory Helicobacteraceae and Campylobacteraceae. Correspondingly, the total SCFAs concentration in the cecum significantly increased by 41.90%, with butyrate and propionate increasing by 23.41% and 45.45%, respectively. PICRUSt2 functional prediction showed that the butyrate biosynthesis pathway (PWY-5677), cofactor metabolism pathways, and carbohydrate degradation pathways related to SCFAs production were significantly upregulated, along with increased abundance of genes encoding ribosomal proteins and ABC transporters, which may be associated with improved microbial metabolic activity and intestinal barrier-related functions.

These findings provide preliminary mechanistic evidence suggesting that creeping fig polysaccharides may alleviate COPD-related pathological changes through modulation of the gut microbiota-SCFAs-lung axis, further expanding their potential relevance in respiratory diseases and highlighting the convergent role of the lung-gut axis in chronic pulmonary conditions. However, PICRUSt2-based inferences regarding microbial metabolic function are derived from 16S rRNA sequences and represent functional predictions rather than direct measurements, warranting validation by metagenomic or metabolomic data. Furthermore, the causal relationships among microbiota changes, SCFAs elevation, and pulmonary inflammation alleviation have yet to be confirmed through fecal microbiota transplantation or SCFAs intervention experiments. Future studies should integrate multi-omics approaches with causal validation models to systematically elucidate the underlying regulatory mechanisms.

### 5.5 Anti-cellular senescence activity

Cellular senescence is closely associated with aging and chronic respiratory diseases, and is commonly characterized by cell-cycle arrest, increased SA-β-gal activity, mitochondrial dysfunction, and oxidative stress (Roger et al., 2021). Recent evidence indicates that creeping fig polysaccharides may alleviate cellular senescence by regulating redox homeostasis. Mo et al. (2026) compared the anti-cellular senescence effects of polysaccharides isolated from creeping fig syconium, stem, and leaf, namely FPP-M, FPSP, and FPLP-M, using cigarette smoke extract (CSE)-induced Beas-2b cells as an *in vitro* model. In this study, 50 μg mL^−1^ CSE was used to induce cellular senescence, and polysaccharides at 25, 50, and 100 μg mL^−1^ were selected for subsequent activity evaluation. Treatment with these polysaccharides improved cell viability, alleviated G0/G1 cell-cycle arrest, restored mitochondrial membrane potential, and modulated the Nrf2/Keap1 signaling pathway. SA-β-gal staining further showed that 50 μg mL^−1^ FPP-M, FPSP, or FPLP-M reduced the number of SA-β-gal-positive senescent cells. Among them, FPLP-M showed the strongest protective effect, as reflected by reduced mitochondrial depolarization, restored G1/S transition, regulation of the Nrf2/Keap1 pathway, and suppression of oxidative stress. The activation of Nrf2 is particularly noteworthy, as this pathway plays a central role in cellular antioxidant defense by regulating the expression of multiple antioxidant and cytoprotective enzymes. Meanwhile, restoration of mitochondrial membrane potential suggests an improvement in mitochondrial function and energy metabolism, which are critical for maintaining cellular homeostasis and delaying the onset of senescence.

These findings suggest that creeping fig polysaccharides, especially FPLP-M, may delay cigarette smoke-induced cellular senescence through Nrf2-mediated redox regulation. Given that oxidative stress and accelerated cellular senescence are important contributors to the progression of chronic respiratory diseases, the protective effects of creeping fig polysaccharides may be relevant to oxidative stress-related airway epithelial injury. However, current evidence is still limited to an *in vitro* bronchial epithelial cell model, and further *in vivo* validation is needed to clarify their anti-senescence potential, the causal involvement of the Nrf2 pathway, and relevance to COPD-related pathological processes.

### 5.6 Hypoglycemic activity

Type 2 diabetes mellitus (T2DM) is characterized by insulin resistance and impaired glucose homeostasis (Krause and De Vito, 2023; Wang et al., 2025). Among the reported biological activities of creeping fig polysaccharides, hypoglycemic activity has received considerable attention due to its potential application in metabolic disease management. Wu et al. (2017) demonstrated that the HG-type pectin FPLP-W isolated from creeping fig syconium exhibited significant hypoglycemic activity in db/db mice. In this study, db/db mice were treated with FPLP-W at 100 and 200 mg kg^−1^·day^−1^ for 4 weeks, with rosiglitazone at 2 mg kg^−1^·day^−1^ as the positive control. FPLP-W effectively reduced fasting blood glucose levels, improved glucose tolerance and insulin sensitivity, and promoted hepatic glycogen accumulation. Mechanistic studies revealed that FPLP-W activated the IRS-1/PI3K/Akt/GSK3β/GS and AMPK/GSK3β/GS signaling pathways, thereby enhancing glycogen synthesis while suppressing the expression of key gluconeogenic enzymes, including glucose-6-phosphatase (G6Pase) and phosphoenolpyruvate carboxykinase (PEPCK). These findings suggest that FPLP-W improves glucose homeostasis through coordinated regulation of hepatic glucose metabolism. Further studies highlighted the importance of polysaccharide fine structures in determining hypoglycemic activity. Chen et al. (2022) prepared a series of pectin derivatives with different degrees of methyl esterification (HG-DMn, DM 3–94) based on FPLP and investigated their activities in insulin-resistant HepG2 cells. In this cell model, HG-DMn was mainly tested at 500 μg mL^−1^ for 24 h, with rosiglitazone (RSG, 2 μM) as the positive control; concentration-dependent assays were also performed for selected samples at 0, 200, 400, 600, 800, and 1,000 μg mL^−1^. The hypoglycemic activity exhibited a non-linear relationship with the degree of methyl esterification, reaching a maximum at DM54. Mechanistic analysis showed that methyl esterification affected the lipophilicity and particle size of pectin, thereby influencing cellular uptake efficiency. Among the tested samples, DM54 possessed the optimal physicochemical properties for intracellular delivery and exhibited the strongest glucose-lowering activity.

These findings indicate that the hypoglycemic effects of creeping fig polysaccharides depend not only on their ability to regulate intracellular metabolic signaling pathways, but also on structural characteristics that determine cellular uptake and bioavailability. In particular, the DM appears to be a critical factor linking pectin structures to biological activities. Taken together, FPLP-W improved glucose homeostasis in db/db mice, while HG-DMn derivatives further demonstrated how methyl esterification may influence glucose consumption through changes in cellular uptake in IR-HepG2 cells. However, the connection between methyl esterification, cellular uptake, bioavailability, and *in vivo* metabolic efficacy remains to be further clarified. Future studies should further clarify the structure–activity relationships of pectin derivatives and evaluate their long-term efficacy and safety, thereby facilitating their potential development as functional food metabolites or therapeutic agents for metabolic disorders.

### 5.7 Immunomodulatory activity

Immunoregulation is a key process to maintain immune homeostasis and defense ability (Josefowicz et al., 2012). Natural polysaccharides are widely considered as potential immunomodulators due to their biocompatibility and low toxicity, especially in the regulation of innate immunity (Murphy et al., 2023; Ying and Hao, 2023). FPW-W, a polysaccharide from the fruit hull of creeping fig, is a type II AG with a Mw of approximately 156 kDa. The main chain is composed of β-1,3-D-Gal, and the side chains are highly branched and rich in Ara (Wu et al., 2020a). *In vitro* experiments showed that FPW-W treatment at 25–400 μg mL^−1^ for 24 h could promote macrophage activation, enhance phagocytosis, and significantly increase the secretion levels of NO, TNF-α, IL-6, and IL-1β with LPS (2 μg mL^−1^) used as the positive control. Notably, NO increased by about 1.97 times at 200 μg mL^−1^, indicating that FPW-W could activate innate immune-related macrophage responses. The immune activity of FPW-W may be closely related to its highly branched AG skeleton. Plant polysaccharides with similar structures (such as Lycium barbarum AG) have been shown to activate immune cells through receptors such as TLR4. Based on this, it is speculated that the β-1,3-D-galactan backbone of FPW-W may form a multivalent binding interface with the highly branched side chains, and cooperate with multiple receptors on the surface of macrophages to amplify the immune signal. However, the specific receptor (TLR4/CR3/Dectin-1) and downstream pathway (NF-κB/MAPK) of FPW-W still lack direct evidence, and the related mechanism is only based on functional association inference, which has not been verified by receptor blocking, gene knockout or signal protein phosphorylation. In addition, the existing evidence is all derived from the *in vitro* macrophage model, and the boundary between “immune enhancement” and “non-specific inflammatory stimulation” cannot be distinguished. Excessive release of pro-inflammatory factors may show a dose-dependent dual effect—moderate activation can enhance defense, excessive release may lead to tissue damage or systemic inflammation. Its safe dose window and long-term risk were not evaluated.

These findings indicate that FPW-W has immunomodulatory potential in macrophage models, but its *in vivo* immune regulatory effects and receptor-mediated mechanisms remain to be clarified. Future studies should integrate receptor blockade, gene silencing, humanized cell models, and animal models of acute and chronic inflammation to systematically elucidate its immunomodulatory mechanisms and safety profile, thereby advancing its transition from an “*in vitro* immune activator” to a candidate molecule with well-defined mechanisms and controllable safety.

## 6 Structure-activity relationships

**FIGURE 7:**
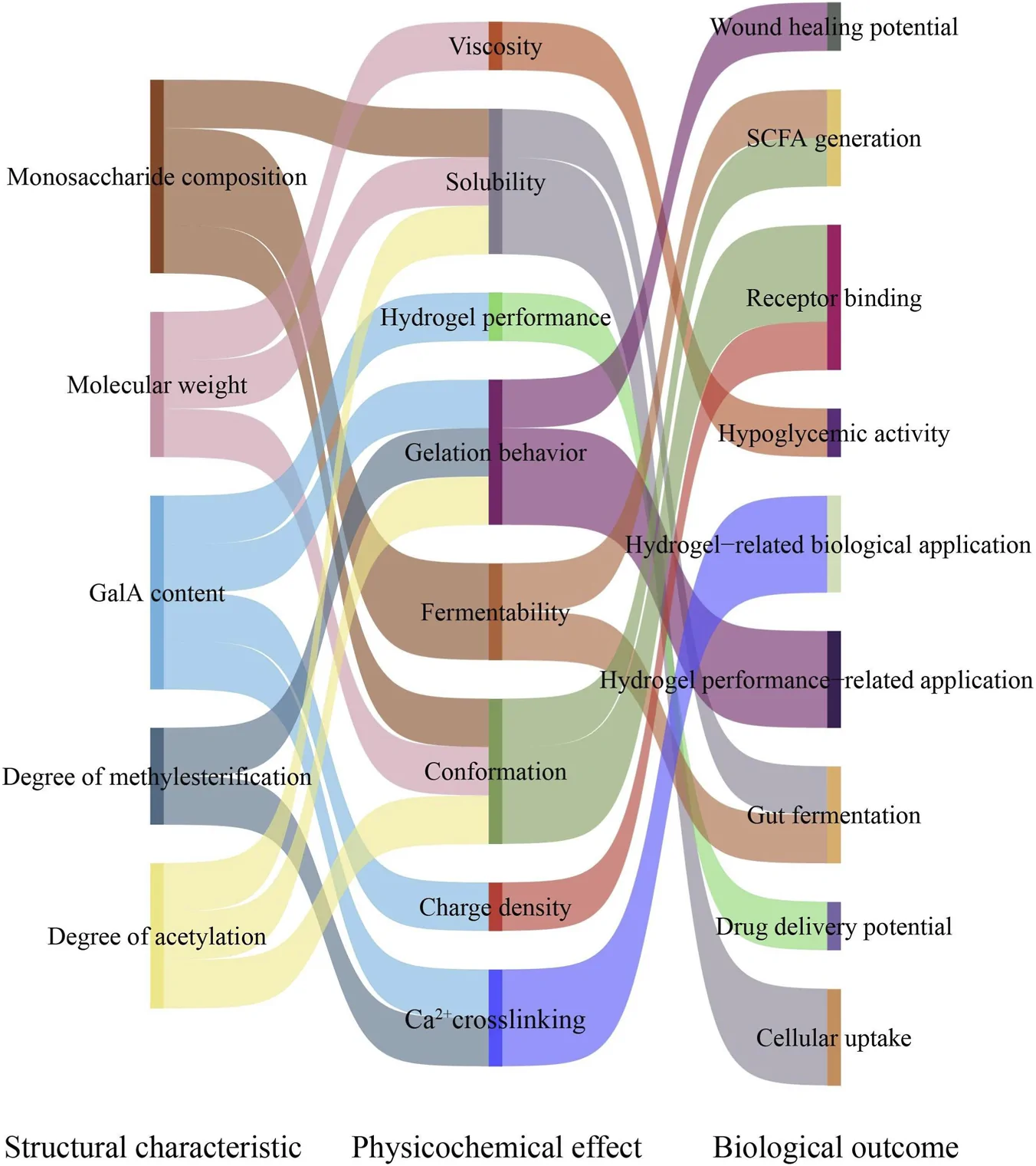
Structural characteristic–physicochemical effect–biological outcome map of creeping fig polysaccharides.

The biological activities of polysaccharides are closely related to their structural characteristics (Ji et al., 2020). Factors such as Mw, monosaccharide composition and chemical structures can affect their activities. Clarifying the structure-activity relationships of creeping fig polysaccharide is helpful to understand its activity mechanism at the molecular level and provide a basis for its directional preparation and functional development. To provide an integrated overview, the potential links among structural characteristics, physicochemical effects and biological outcomes are summarized in Figure 7. In this section, combined with the existing research, the structure-activity relationships of creeping fig polysaccharide are analyzed from three aspects: Mw, monosaccharide composition and chemical structures.

Mw is one of the important factors affecting the biological activities of polysaccharides (Chen et al., 2017). Polysaccharides with different Mw may exhibit different activities (Wei et al., 2018). The high Mw polysaccharide FPP-M (966.70 kDa) showed anti-cellular senescence activity (Mo et al., 2026), while the low Mw FPLP-W (34.69 kDa) had an anti-obesity activity (Wu et al., 2020b). In general, Mw can regulate the activities of polysaccharides by affecting their solubility, chain conformation and their interaction with receptors or cells. When the Mw is high, increased chain entanglement, solution viscosity, and steric hindrance may limit molecular diffusion, cellular uptake, and receptor binding. When the Mw is low, the polysaccharide may show improved solubility and accessibility, but it may not be sufficient to maintain the spatial conformation required for certain activities. Therefore, In-depth analysis of the corresponding relationship between the Mw and activity of creeping fig polysaccharide, and clarifying the appropriate Mw range for different activities will help to achieve directional regulation of its activity by means of controllable degradation.

The monosaccharide composition and uronic acid content have important effects on the activity of creeping fig polysaccharide. GalA -rich pectin polysaccharides from creeping fig generally showed strong biological activities. For example, the syconium polysaccharides FPP-Y and FPLP, as well as the achene pectin FPLP-W showed anti-allergic asthma, hypoglycemic and anti-obesity activities, respectively (Ye et al., 2024; Wu et al., 2017; Wu et al., 2020b), suggesting that GalA is an important structural basis for the activity of creeping fig pectin polysaccharides. The high GalA and uronic acid contents can increase charge density and hydration capacity, and may further influence solubility, molecular conformation, Ca^2+^ binding, gut fermentation and interactions with cells or proteins. Among the neutral polysaccharides, FPW-W is AG-II rich in Ara and Gal, showing significant immunomodulatory activity, which can activate macrophages and promote the release of NO and various cytokines, which is consistent with the general immunomodulatory activity of arabinogalactan polysaccharides (Wu et al., 2020a). It can be seen that the difference in monosaccharide composition gives creeping fig polysaccharides different activity tendencies: acidic pectic polysaccharides are mostly related to metabolic regulation and anti-inflammatory activity, while neutral arabinogalactan has more advantages in immune regulation.

Among the structural characteristics of creeping fig polysaccharides, the DM of pectin has the most systematic and clear influence on its biological activities. Chen et al. (2022) prepared a series of samples with esterification degree gradient (DM3 to DM94) based on the same pectin skeleton and investigated their hypoglycemic activity. The results showed that with increasing DM, the hypoglycemic activity and cellular uptake of polysaccharides first increased and then decreased, reaching a maximum at a DM of approximately 54%. Mechanism studies have shown that the DM affects the uptake of polysaccharides by cells by regulating the pinocytosis of cells and the endocytosis process mediated by caveolins, thereby determining their activity (Chen et al., 2022). These results showed that an appropriate DM is more favorable for the activity of creeping fig pectin than extremely low or high DM. In addition to the DM, DA also affects its activity. For example, in the study of anti-colitis, samples with different degrees of acetylation showed significant differences in regulating inflammatory factors and intestinal flora (Chen et al., 2024). In addition, the DM can also affect the gel behavior by changing the carboxyl distribution and molecular aggregation state of pectin. For example, WEP-0.3 and WEP-0.4 with low DM exhibit porous sponge-like and dense rod-like structures on the microstructure, respectively (Wang et al., 2019). These findings suggest that fine chemical structures, especially DM and DA, are key factors regulating the physicochemical behavior, biological activity and functional performance of creeping fig polysaccharides.

In summary, the biological activities of creeping fig polysaccharide are regulated by Mw, monosaccharide composition and chemical structures. Mw affects its chain conformation and interaction with cells. Monosaccharide composition (especially GalA content) determines its activity tendency, while the DM finely regulates its activity. Among them, the research on the fine structure represented by esterification degree is the most in-depth, which provides an important basis for the directional modification and functional optimization of creeping fig pectin. Further elucidating the synergistic effect between various structural factors and the mechanism of action at the molecular level will be an important direction for the study of the structure-activity relationships of creeping fig polysaccharide, and it is expected to achieve the directional design of its function through the precise regulation of the structures.

## 7 Hydrogel properties of creeping fig polysaccharides and their applications in medicine

**FIGURE 8:**
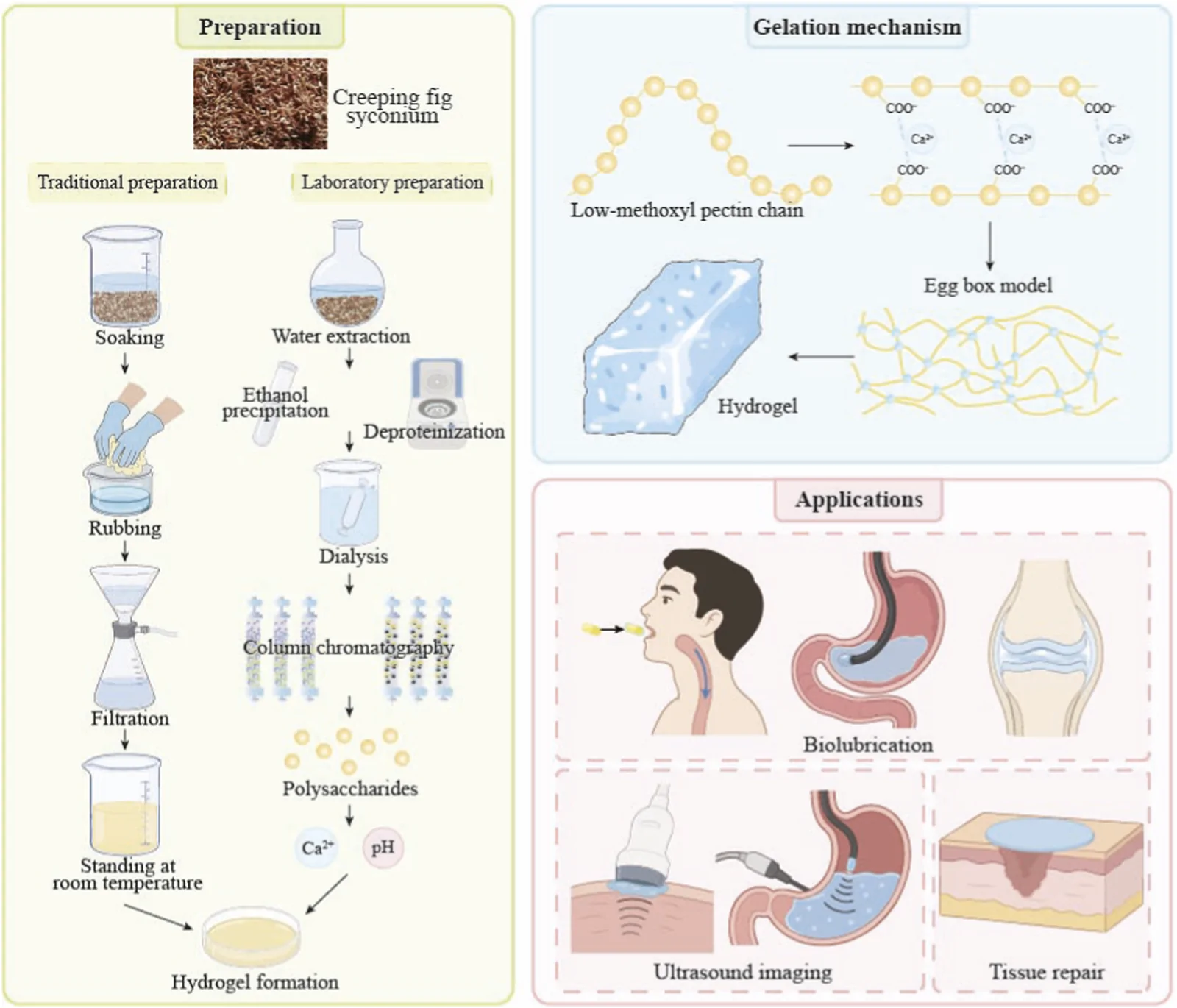
Preparation, gelation mechanism, and medical applications of creeping fig polysaccharide hydrogels, including tissue repair, biolubrication, and ultrasound imaging.

Hydrogel is a kind of polymer material with three-dimensional network structure, which can hold a large amount of water (Najihah et al., 2024). Creeping fig achene pectin can spontaneously form a gel at room temperature, which is its most distinctive functional property. Figure 8 illustrates the preparation, gelation mechanism, and medical applications of creeping fig polysaccharide hydrogels.

### 7.1 Preparation, gelation mechanism and structural characteristics of creeping fig polysaccharide hydrogels

Creeping fig polysaccharide hydrogels represent an important functional feature that distinguishes creeping fig from most plant polysaccharide resources. In traditional preparation, mature achenes are washed, soaked and rubbed, so that the surface mucilage is released into the water phase, filtered to remove impurities, and allowed to form a transparent or translucent gel at room temperature (Liang et al., 2012). Unlike most polysaccharide hydrogels that require exogenous cross-linking agents, heating or modification, creeping fig hydrogels are formed under mild conditions, mainly relying on the hydration, chain expansion and cross-linking of pectin polysaccharides. In the experimental study, the achenes of creeping fig were usually extracted by water, precipitated by alcohol, deproteinized, dialyzed and purified by column chromatography to obtain the polysaccharide metabolites with clear composition, and then the hydrogels were constructed by adjusting the polysaccharide concentration, Ca^2+^ concentration, pH and temperature (Chen et al., 2021). Both traditional preparation and laboratory preparation showed that the formation of creeping fig polysaccharide hydrogels was closely related to its pectin polysaccharide and ion crosslinking behavior.

The formation mechanism of creeping fig polysaccharide hydrogels is mainly related to the structure of low methoxyl pectin. Creeping fig pectin polysaccharide is rich in GalA residues, and the molecular chain contains a large number of carboxyl groups. After ionization to—COO^−^, the carboxyl group can be coordinated with Ca^2+^ to form an ion bridge between adjacent pectin chains (Liang et al., 2012). This process is often explained by the “egg-box” model, that is, Ca^2+^ is embedded between the pectin chains and forms an ordered cross-linking zone with the carboxyl group. With the increase of crosslinking sites, the dispersed polysaccharide chains aggregate to construct a continuous three-dimensional network, and the water molecules are limited in the pores, thus forming hydrogels with certain mechanical strength and water retention capacity. In addition, hydrogen bond, chain entanglement, electrostatic interaction, polysaccharide concentration and methoxylation degree also affect the formation and stability of the network. The low-methoxyl structure provides the structural basis for Ca^2+^-mediated gelation, but the resulting gel properties are not determined by DM alone. In pectin-based materials, high-methoxyl pectin may show better cohesive or flexural properties under certain conditions, indicating that different pectin structures may favor different mechanical behaviors (Byun et al., 2019). Therefore, molecular weight, GalA content, DA, methyl-ester distribution, Ca^2+^ concentration, water content and formulation conditions should be considered together when evaluating the gel strength, flexibility and stability of creeping fig polysaccharide hydrogels.

In terms of structure, creeping fig polysaccharide hydrogels belong to a typical three-dimensional network system with high water content. After freeze-drying, it is mostly porous structure, which is conducive to water storage, material diffusion and stress buffering (Wang et al., 2019). The hydroxyl and carboxyl groups on the chain can bind water molecules through hydrogen bonds, giving it better water retention capacity. It should be pointed out that the current systematic rheological characterization of its viscoelasticity is mostly based on the composite gel system constructed by creeping fig polysaccharide and starch. When the storage modulus G′ is higher than the loss modulus G″ in this kind of system, it shows that the elastic behavior is dominant and a stable network has been formed (Pan et al., 2024). These results can provide a reference for understanding the gelation behavior of creeping fig polysaccharide, but the viscoelasticity and rheological characteristics of pure creeping fig polysaccharide hydrogels need to be systematically determined. In general, its high water content, porous structure and water retention capacity constitute an important material basis, and also provide a basis for its potential applications in medical fields such as tissue repair, biolubrication and ultrasound imaging.

### 7.2 The application prospect of creeping fig polysaccharide hydrogels in the field of medicine

Creeping fig achene pectin can form a hydrogel with water retention and viscoelasticity by Ca^2+^ crosslinking. At the same time, creeping fig polysaccharide can inhibit the expression of pro-inflammatory factors in colitis model, and has the activities of immune regulation, regulation of intestinal flora and delaying cell senescence (Chen et al., 2024; Mo et al., 2026). The combination of material properties and multiple biological activities makes it have potential value in the fields of tissue repair, biolubrication and ultrasound imaging (Sun et al., 2022).

In terms of tissue repair, hydrogels can provide moist coverage and interface protection (Zhao et al., 2025; Mi et al., 2026). Alginate and commercial pectin hydrogels have been widely explored as wound dressings or local drug delivery matrices, but their performance usually depends on controlled crosslinking, formulation optimization or combination with other functional metabolites (Cheong et al., 2025). Miyazaki et al. (2004) also reported that jelly fig extract from *Ficus awkeotsang* could serve as a hydrophilic matrix for controlled theophylline release, providing an early example of pharmaceutical matrix design using pectin-rich gel-forming *Ficus* materials. For creeping fig hydrogels, hydroxyl and carboxyl groups can bind water through hydrogen bonding, while the Ca^2+^-crosslinked network may reduce water migration, prolong local residence time and form a soft protective layer. Together with the reported inhibitory effects of creeping fig polysaccharides on pro-inflammatory factors and cellular senescence, these properties provide a rationale for wound-related exploration. However, wound repair models using creeping fig polysaccharide hydrogels have not yet been reported. Future studies should evaluate water retention, moisture permeability, adhesion, cytocompatibility, inflammatory responses, collagen deposition and wound closure. The potential of biolubrication is mainly related to interface hydration, network stability and deformation response (Adibnia et al., 2021). Cellulose derivatives and konjac glucomannan-based hydrogels have been explored as hydrated polymer networks for tissue-contacting or lubricating materials, but they often require chemical modification, composite formulation or additional crosslinking to balance spreading, retention and mechanical stability (Lopes et al., 2011). Creeping fig polysaccharide hydrogels may provide another hydrated pectic network, in which hydrophilic groups bind water to form a hydration layer and the three-dimensional gel network helps maintain interfacial continuity. Their viscoelasticity also suggests possible spreading and deformation adaptability (Pan et al., 2024). Therefore, they may be explored as edible swallowing aid gels, solid drug surface lubricating layers or endoscopic lubricating media. Joint cavity lubrication is a more distant direction because it requires injectability, cyclic loading stability, cartilage compatibility, *in vivo* retention and long-term safety (He et al., 2025). Lubrication performance should be verified by friction coefficient, lubricating film stability, mucosal friction, structural recovery and cyclic loading tests. In ultrasonic medicine, creeping fig polysaccharide hydrogels may be explored as surface ultrasound coupling media or oral gastrointestinal ultrasound-assisted imaging media (Nam et al., 2020). Cellulose-based oral ultrasound contrast agents have been investigated for gastric ultrasound because hydrated polysaccharide systems can fill the gastric cavity, reduce gas interference and form an acoustic window (Sui et al., 2021). For creeping fig polysaccharide hydrogels, the continuous aqueous phase may help fill small gaps between the probe and skin, while the gel state may maintain interfacial continuity during probe movement. Their edible gel background also provides a possible advantage for oral gastrointestinal use. Compared with clear water, a creeping fig polysaccharide system with appropriate viscosity or weak gel characteristics may improve gastric spreading and retention. However, excessive gel strength may cause uneven distribution, bubble retention or delayed gastric emptying. This direction is therefore more suitable for flowable viscous sols or weak gels, and future studies should evaluate sound velocity, acoustic impedance, attenuation coefficient, transmittance, bubble content, swallowing ability, gastric distribution and gastric emptying.

In general, creeping fig polysaccharide hydrogels are promising for biomedical exploration. However, their current medical application evidence remains limited. Existing studies have not directly tested creeping fig polysaccharide hydrogels in wound repair, biolubrication or ultrasound-assisted imaging models, and there is still no *in vivo* or clinical evidence for these application scenarios. Key limitations also include insufficient formulation standardization, unclear relationship between crosslinking degree and material performance, lack of mechanical and tribological evaluation, lack of acoustic characterization, uncertain sterilization and storage stability, and incomplete biosafety assessment. Therefore, these applications should be viewed as forward-looking research directions rather than established medical uses. Future work should optimize composition, crosslinking degree and gel strength according to specific application requirements, and establish systematic biological, mechanical, tribological, acoustic and safety evaluation systems. Such studies would help clarify whether creeping fig polysaccharide hydrogels can move from laboratory material research toward practical biomedical development.

## 8 Challenges and future perspectives

In recent years, studies on creeping fig polysaccharides have gradually increased, but this field is still at a relatively early stage. Current evidence mainly focuses on extraction and separation, structural characterization, *in vitro* experiments and animal model evaluation, and there is still a considerable gap between existing studies and the clarification of pharmacological mechanisms and practical application translation. In terms of extraction, the preparation process of creeping fig polysaccharides has not yet been standardized. Differences in raw material sources, harvest periods, extraction solvents, extraction temperature, ethanol precipitation conditions, deproteinization methods and purification processes among different studies may lead to marked variations in yield, molecular weight, monosaccharide composition, uronic acid content, DM and degree of acetylation. Therefore, more standardized extraction, purification and quality control procedures should be established in the future, and batch-to-batch consistency should be further evaluated to provide a reliable basis for subsequent structural analysis, biological activity comparison and application development.

The structural characteristics of creeping fig polysaccharides also require further investigation. Most current studies mainly focus on molecular weight, monosaccharide composition, uronic acid content and basic structural information obtained by FT-IR and NMR, whereas their fine structural characteristics remain insufficiently understood. For example, the backbone linkage patterns, side-chain distribution, methyl-ester distribution pattern, acetyl group position, higher-order conformation and chain aggregation behavior in solution still lack systematic investigation. For pectic polysaccharides, the overall DM alone cannot fully explain differences in gel formation ability and biological activities, because pectins with similar DM values may still differ in the distribution of methyl esters along the GalA backbone. In recent studies on plant-derived pectins, enzymatic fingerprinting combined with HILIC-FLR-MSn has been used to determine methyl-esterification patterns in HG-rich pectin fractions from different plant sources, and HILIC-ELSD/ESI-MSn has also been applied to characterize methyl ester and acetyl group distributions in sugar beet pectin-derived oligomers (Yu et al., 2022; Remoroza et al., 2012). These studies provide useful methodological references for future fine-structural analysis of creeping fig pectic polysaccharides. Therefore, future studies may combine enzymatic fingerprinting, HILIC-FLR-MSn or related HILIC-MS-based methods with multidimensional NMR, SEC-MALLS, AFM and molecular simulation to further clarify the fine structural characteristics of creeping fig polysaccharides and their relationships with physicochemical properties and biological activities.

The evidence level of current studies on biological activities remains limited. Creeping fig polysaccharides have been reported to possess hypoglycemic, anti-colitis, anti-obesity, immunomodulatory and anti-cellular senescence activities, but most evidence is derived from *in vitro* experiments or animal models, with a lack of clinical support. Some mechanistic explanations are still mainly based on correlative results, such as changes in inflammatory factors, oxidative stress indicators, gut microbiota composition and SCFA levels, which are not sufficient to fully demonstrate causal relationships. Future studies should pay more attention to the rigor of experimental design, including clear information on polysaccharide fractions, dose range, administration route, treatment duration, positive and negative controls, minimal effective dose and toxicity observations. For gut microbiota-related mechanisms, future studies should not remain limited to correlation analyses of microbial composition and SCFA levels, but should further clarify whether changes in gut microbiota directly participate in the biological activities of creeping fig polysaccharides through microbiota intervention, metabolite supplementation or key pathway blocking.

Studies on the pharmacokinetics, bioavailability and safety of creeping fig polysaccharides are also clearly insufficient. As macromolecular polysaccharides, whether they are directly absorbed after oral administration, how they are degraded in the gastrointestinal tract, whether they mainly exert biological activities through gut microbiota fermentation, and how their active fragments or metabolites participate in host regulation remain unclear. Meanwhile, systematic evaluations are still lacking regarding the conversion of animal doses to human doses, long-term intake safety, potential immunogenicity, intestinal tolerance and interactions with other drugs or food metabolites. In the future, studies on acute and long-term toxicity, pharmacokinetics, intestinal metabolism, biodistribution and dose conversion should be carried out to provide a more reliable safety basis for the development of functional foods, adjuvant pharmacological interventions and biomedical materials.

In terms of application translation, research on creeping fig polysaccharide hydrogels is still at an early stage. Existing studies mainly provide preliminary evidence for their gel formation ability, water-holding properties and pectic structural basis, which support further exploration of their potential applications in tissue repair, biolubrication and ultrasound-assisted imaging. However, different application scenarios have distinct requirements for material performance and biosafety. Therefore, systematic performance evaluation and safety validation should be established for specific applications. For tissue repair, water absorption, water retention, moisture permeability, wound adhesion, cytocompatibility, inflammatory responses, collagen deposition and wound closure should be further investigated. For biolubrication, the friction coefficient, lubricating film stability, dehydration resistance, structural recovery under cyclic loading and tissue compatibility should be evaluated. For ultrasound-assisted imaging, acoustic velocity, acoustic impedance, acoustic attenuation, sound transmission, bubble residues, interfacial stability and actual imaging quality should be examined. In addition, sterilization methods, storage stability, large-scale preparation, quality control and regulatory pathways of hydrogel products also need to be further clarified.

## 9 Conclusion and discussion

Creeping fig is widely distributed and rich in resources, and has long been used as a traditional edible plant. The spontaneous gelation of its achene pectin at room temperature is its most distinctive functional property, and the polysaccharide is one of its important functional metabolites. In this paper, the extraction, purification, structural characteristics, biological activities, structure-activity relationships, and hydrogel properties of creeping fig polysaccharide were systematically reviewed, and its application potential in the fields of medicine, such as tissue repair, biolubrication, and ultrasound imaging, was summarized. By linking the structural characteristics of creeping fig polysaccharide with its functional activities, this paper aims to systematically summarize the current research status, clarify the material basis of its functions, and provide a reference for its further development in the fields of functional food and medicine.

The achene and syconium polysaccharides of creeping fig are mostly pectin polysaccharides rich in GalA. The low methoxyl pectin of the achene can be cross-linked by calcium ions and spontaneously gelled at room temperature, which is the structural source of creeping fig gel properties. At present, the analysis of this kind of pectin mostly stays in the overall parameter of esterification degree. However, the esterification degree only reflects the overall proportion of methyl ester groups, and it is difficult to describe the arrangement of ester groups along the main chain of GalA. In the calcium ion cross-linking of low methoxyl pectin, only continuously arranged free carboxyl groups can form stable cross-linking zones. Therefore, even at the same esterification degree, when the ester groups are block-distributed or randomly distributed, the cross-linking zones thus formed differ significantly in length and continuity, which affects the compactness and uniformity of the gel network. This effect has been indirectly reflected in creeping fig pectin: WEP-0.3 (DM = 10.46%) and WEP-0.4 (DM = 6.67%), both belonging to low methoxyl pectin, show porous sponge-like and dense rod-like microstructures, respectively, indicating that changes in ester group characteristics can significantly alter the molecular aggregation mode of pectin, and the distribution mode of ester groups is likely to be a key factor that has not yet been identified. Therefore, the DM alone is not sufficient to reveal the gelation mechanism of creeping fig pectin. In the future, the distribution of ester groups along the main chain can be determined by partial enzymatic hydrolysis with pectin lyase combined with mass spectrometry fingerprint analysis, and correlated with gel strength, network morphology and other performance indicators, so as to clarify the gelation mechanism of creeping fig pectin at the molecular level.

The study on the activity of creeping fig polysaccharides has shown certain characteristics, especially the fine regulation of pectin esterification degree on its hypoglycemic activity, which provides a systematic example for understanding the structure-activity relationships of polysaccharides. Studies have shown that as the DM increases from 3% to 94%, the lipophilicity of pectin gradually increases (logP increases from −1.253 to about −0.321), and the hydrodynamic particle size changes (Chen et al., 2022). Its hypoglycemic activity and cell uptake efficiency reach their highest when the DM is about 54%, showing a significant non-monotonic relationship. This phenomenon suggests that the DM may affect the way and efficiency of pectin uptake by cells by changing the lipophilicity and particle size of pectin, and then determine the activity strength. However, the regulation of the esterification degree in this process still needs to be further analyzed at the cellular level. Whether pectin with different degrees of esterification enters cells through pinocytosis or caveolin-mediated endocytosis, whether the dominant pathway shifts along the esterification gradient, and the resulting differences in intracellular transport currently remain to be clarified due to a lack of direct evidence; the way it regulates insulin signaling pathways such as IRS-1/PI3K/Akt after uptake also needs to be further elucidated. In the future, the main endocytosis pathways of pectin with different esterification degrees into cells can be distinguished one by one by means of clathrin- and caveolin-specific endocytosis inhibitors and caveolin gene silencing. Combined with the detection of intracellular signal molecule expression and activation levels, the internal relationship between esterification degree, cell uptake, and hypoglycemic activity can be clarified, providing a mechanistic basis for the precise structural design of creeping fig pectin.

Creeping fig polysaccharide is naturally edible, can spontaneously form a gel at room temperature, and has certain anti-inflammatory activity, which has application value in the field of food and medicine. In the food field, it can be used as a natural gelling agent, thickener, and dietary fiber source. In the field of medicine, it is expected to be used for tissue repair, biolubrication, and ultrasound imaging. However, to translate these potentials into practical products, the problem of unstable gel properties needs to be solved first. The gelation ability of creeping fig pectin depends on the DM and the content of endogenous calcium ions, and the pectin obtained from different sources and preparation methods has obvious differences in these two indicators, so the gel properties fluctuate greatly between batches. The improvement of this problem depends on the standardization of raw material classification and the extraction process, and the controllable adjustment of the esterification degree and calcium ion content. On the basis of stable performance, it can also compensate for the lack of mechanical strength of natural hydrogels by compounding with other natural polymers to meet the needs of load-bearing scenarios such as dressings and lubrication (Hu and Xu, 2020; Lee et al., 2023). In addition, although creeping fig has a long-term food safety foundation, its toxicology and biosafety evaluation as a medical material still need to be systematically supplemented, which is an important prerequisite for its clinical application.

In summary, creeping fig polysaccharide has shown important development value in the field of functional food and medicine due to its unique gel properties, diverse biological activities and natural edible safety basis. However, in order to give full play to its potential, it is still necessary to continue to deepen the fine structure analysis, activity mechanism elucidation and application transformation. With the advancement of research, creeping fig polysaccharide is expected to develop from traditional edible resources to high-value natural products with both nutrition and function.

## Glossary

Ara: Arabinose
COPD: Chronic obstructive pulmonary disease
CSE: Cigarette smoke extract
DA: Degree of acetylation
DM: Degree of methylesterification
DSS: Dextran sulfate sodium
DAO: Diamine oxidase
FT-IR: Fourier transform infrared spectroscopy
Fuc: Fucose
Gal: Galactose
GalA: Galacturonic acid
GC-MS: Gas chromatography-mass spectrometry
Glc: Glucose
G6Pase: Glucose-6-phosphatase
HPGPC: High performance gel permeation chromatography
HG: Homogalacturonan
HPAEC: Ion chromatography
LDL-C: Low-density lipoprotein cholesterol
MDA: Malondialdehyde
Man: Mannose
Mw: Molecular weight
MPO: Myeloperoxidase
NMR: Nuclear magnetic resonance
OVA: Ovalbumin
PEPCK: Phosphoenolpyruvate carboxykinase
Rha: Rhamnose
SCFAs: Short-chain fatty acids
SOD: Superoxide dismutase
TC: Total cholesterol
T2DM: Type 2 diabetes mellitus
AG-II: Type II arabinogalactan
Xyl: Xylose
